# Hematopoietic stem cells undergo a lymphoid to myeloid switch in early stages of emergency granulopoiesis

**DOI:** 10.15252/embj.2023113527

**Published:** 2023-10-17

**Authors:** Karolina Vanickova, Mirko Milosevic, Irina Ribeiro Bas, Monika Burocziova, Asumi Yokota, Petr Danek, Srdjan Grusanovic, Mateusz Chiliński, Dariusz Plewczynski, Jakub Rohlena, Hideyo Hirai, Katerina Rohlenova, Meritxell Alberich‐Jorda

**Affiliations:** ^1^ Laboratory of Hemato‐oncology Institute of Molecular Genetics of the Czech Academy of Sciences Prague Czech Republic; ^2^ Faculty of Science Charles University Prague Czech Republic; ^3^ Institute of Biotechnology of the Czech Academy of Sciences Prague Czech Republic; ^4^ Laboratory of Stem Cell Regulation, School of Life Sciences Tokyo University of Pharmacy and Life Sciences Tokyo Japan; ^5^ Laboratory of Bioinformatics and Computational Genomics, Faculty of Mathematics and Information Science Warsaw University of Technology Warsaw Poland; ^6^ Laboratory of Functional and Structural Genomics, Centre of New Technologies University of Warsaw Warsaw Poland; ^7^ Childhood Leukaemia Investigation Prague, Department of Pediatric Haematology and Oncology, 2^nd^ Faculty of Medicine, University Hospital Motol Charles University in Prague Praha Czech Republic

**Keywords:** CD201, emergency granulopoiesis, lymphoid‐biased HSC, myeloid‐biased HSC, Development, Haematology, Stem Cells & Regenerative Medicine

## Abstract

Emergency granulopoiesis is the enhanced and accelerated production of granulocytes that occurs during acute infection. The contribution of hematopoietic stem cells (HSCs) to this process was reported; however, how HSCs participate in emergency granulopoiesis remains elusive. Here, using a mouse model of emergency granulopoiesis we observe transcriptional changes in HSCs as early as 4 h after lipopolysaccharide (LPS) administration. We observe that the HSC identity is changed towards a myeloid‐biased HSC and show that CD201 is enriched in lymphoid‐biased HSCs. While CD201 expression under steady‐state conditions reveals a lymphoid bias, under emergency granulopoiesis loss of CD201 marks the lymphoid‐to‐myeloid transcriptional switch. Mechanistically, we determine that lymphoid‐biased CD201^+^ HSCs act as a first response during emergency granulopoiesis due to direct sensing of LPS by TLR4 and downstream activation of NF‐κΒ signaling. The myeloid‐biased CD201^−^ HSC population responds indirectly during an acute infection by sensing G‐CSF, increasing STAT3 phosphorylation, and upregulating LAP/LAP* C/EBPβ isoforms. In conclusion, HSC subpopulations support early phases of emergency granulopoiesis due to their transcriptional rewiring from a lymphoid‐biased to myeloid‐biased population and thus establishing alternative paths to supply elevated numbers of granulocytes.

## Graphical abstract

A mouse model of emergency granulopoiesis provides insight into the role of hematopoietic stem cells in the enhanced and accelerated production of granulocytes that occurs during acute infection.

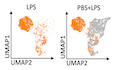

## Introduction

Granulocytes represent an essential part of the innate immune system and serve as major effector cells responsible for the control of bacterial and fungal pathogens (Nauseef & Borregaard, 2014). In steady‐state conditions, the production of granulocytes is known as granulopoiesis, while during severe infections emergency granulopoiesis is taking place. Compared to steady‐state, emergency granulopoiesis is differentially regulated at the transcriptional level (Hirai *et al*, 2006) and requires appropriate expression of cell‐ and stage‐specific transcription factors. The response is initiated by pathogen sensing, which occurs by two distinct mechanisms. Direct pathogen sensing relies on the expression of appropriate pathogen recognition receptors (PRRs) directly on the hematopoietic stem and progenitor cells (HSPCs; Zhao *et al*, 2014; Takizawa *et al*, 2017). Indirect pathogen sensing relies on the recognition of pathogens by PRRs on the surface of immune cells or cells of the bone marrow (BM) niche, which in turn secrete high levels of G‐CSF that are recognized by G‐CSF receptor (G‐CSF‐R) on the surface of HSPCs (Boettcher *et al*, 2014). As a consequence, proliferation and differentiation of myeloid progenitors is rapidly enhanced and *de novo* generation of granulocytes occurs (Manz & Boettcher, 2014). At the progenitor level, this is mostly orchestrated by the switch from C/EBPα‐ to C/EBPβ‐mediated transcription (Hirai *et al*, 2006; Satake *et al*, 2012). Interestingly, individual C/EBPβ isoforms have also been shown to differentially regulate HSC fate during hematopoietic regeneration (Sato *et al*, 2020). First, LIP isoform is upregulated and facilitates proliferation, while the later, LAP/LAP* isoforms enable myeloid differentiation of the HSPCs. Despite our vast knowledge of the physiological changes that occur at the level of committed progenitors and granulocytes during emergency granulopoiesis, the mechanism through which the emergency granulopoiesis response is supported by the most immature hematopoietic stem cells (HSCs) remains unclear.

The hematopoietic system was originally described as a hierarchical and linear model, in which multipotent HSCs reside at the apex of the hematopoietic tree. In this model, HSCs have the ability to self‐renew as well as to commit towards myeloid or lymphoid progenitors, responsible for coordinating the production of mature blood cells (Seita & Weissman, 2010). However, recent advances in the field showed that certain degree of lineage commitment already exist at the level of HSCs (Hirschi *et al*, 2017), accounting for the existence of myeloid‐, lymphoid‐, and platelet‐biased HSCs (Benz *et al*, 2012; Sanjuan‐Pla *et al*, 2013; Chen *et al*, 2017; Montecino‐Rodriguez *et al*, 2019). Nevertheless, how strong the bias is, whether it is context‐dependent, and whether these HSCs can be rewired to different lineage remains largely unknown. Recent reports suggest that distinct HSC subpopulations can directly enhance cell production under certain conditions (Haas *et al*, 2015; Pietras *et al*, 2016; Singh *et al*, 2018; Rommel *et al*, 2022). Haas and colleagues reported the existence of an HSC fraction characterized by stem‐like megakaryocyte‐committed progenitor features. These particular HSCs are quiescent during steady‐state megakaryopoiesis; however, they get primed during acute inflammation and provide efficient platelet recovery (Haas *et al*, 2015). Similarly, it was reported that HSCs, but not MPPs, drive erythropoiesis during chronic erythroid stress (Singh *et al*, 2018). Despite numerous studies showing that HSCs and MPPs respond directly to bacterial pathogens through toll‐like receptors (Nagai *et al*, 2006; Sioud *et al*, 2006; Schuettpelz *et al*, 2014; Herman *et al*, 2016; de Laval *et al*, 2020), our knowledge of the early molecular changes driving the switch from steady‐state to emergency granulopoiesis in the most immature populations is missing.

Here, we show that murine HSCs respond to emergency granulopoiesis shortly after infection by transcriptionally rewiring specific subpopulations, expanding the myeloid‐biased HSCs, and favoring the production of myeloid progenitors. We identify CD201 as a cell surface marker that allows the separation of HSCs to distinct lineage bias, and report that during emergency granulopoiesis HSCs compromise the lymphoid output and favor the myeloid production. We demonstrate that CD201^+^ and CD201^−^ HSCs sense acute infections by using distinct receptors, activating distinct signaling pathways, and ultimately employing distinct isoforms of the transcription factor C/EBPβ. Altogether, we provide cellular and molecular insights into the regulation of emergency granulopoiesis at the stem cell level and point to the central role and considerable flexibility of the HSCs pool during emergency conditions, providing a deeper understanding of HSC biology.

## Results

### HSCs transcriptionally respond to emergency granulopoiesis at early stages

**Figure 1:**
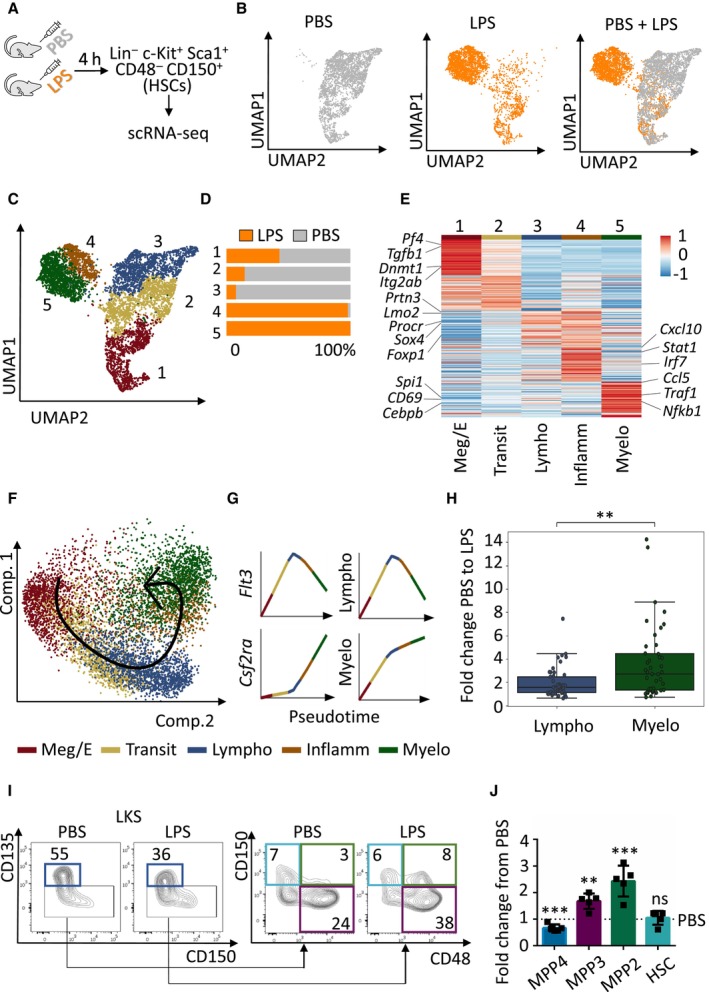
HSCs respond to emergency granulopoiesis AGraphical representation of the experimental design. WT C57BL/6 mice were injected with PBS vehicle control or LPS (35 μg) to induce emergency granulopoiesis. Four hours after injection Lin^−^ c‐Kit^+^ Sca‐1^+^ CD48^−^ CD150^+^ HSCs were sorted and subjected to scRNA‐seq.BUMAP plots color‐coded for HSCs isolated from PBS‐treated (left), LPS‐treated (middle) mice and overlap (right).CUMAP plot of LPS‐ and PBS‐treated transcriptomes color‐coded for the 5 phenotypes identified by graph‐based clustering.DRelative contribution of PBS‐ (gray) and LPS (orange)‐treated HSCs to each cluster. *X*‐axis indicates percentage.EGene expression levels of top‐ranking marker genes for each cluster.F, GPseudotime trajectory of the indicated HSC phenotypes (F) and Loess regression‐smoothened gene expression of the indicated genes in pseudotime (G).HBoxplot showing the enrichments of the ATAC‐seq near‐TSS peaks *q*‐values of top‐ranking marker genes from (E). Data shown as fold change from PBS to LPS. In the boxplot, central bands indicate median, boxes indicate1^st^ and 3^rd^ quartiles, and whiskers indicate furthest data point within 1.5 times the interquartile range (defined as the difference between 3^rd^ and 1^st^ quartile) from the appropriate quartile. Welch's *t*‐test independent samples with Bonferroni correction was used to assess statistical significance (**P < 0.01).IRepresentative flow cytometry plots of multipotent progenitors in the BM cells isolated from mice treated with PBS control or LPS for 4 h, numbers indicate percentages of LKS. Dark blue boxes indicate multipotent progenitor 4 (MPP4), violet boxes indicate MPP3, green boxes indicate MPP2, and light blue boxes indicate HSCs.JQuantification of panel (I). *Y*‐axis indicates fold change from PBS‐treated mice to LPS‐treated mice. At least five animals were included in each group. Data represent mean ± SD from two independent experiments. Two‐tailed Student's *t*‐test was used to assess statistical significance (**P < 0.01, ***P < 0.001, ns, not significant). Source data are available online for this figure.

Since recent reports suggest that a fraction of HSCs can be activated in the context of stress (Haas *et al*, 2015; Singh *et al*, 2018), we investigated whether HSCs respond during emergency granulopoiesis. We challenged WT mice with lipopolysaccharide (LPS) or vehicle control PBS, and determined 4 h as the early time point when emergency granulopoiesis is initiated and induces early transcriptional changes in HSCs without altering HSC numbers or expression of HSC surface markers (Appendix Fig S1A–D). Thus, HSCs were sorted 4 h after the challenge and subjected to single cell RNA sequencing (scRNA‐seq; Fig 1A). Interestingly, the HSC transcriptional profile in PBS versus LPS was dramatically affected (Fig 1B) and allowed us to identify five distinct HSC identities in PBS and LPS conditions (Fig 1C and D). This distribution was defined by specific gene expression patterns (Appendix Table S1), and divided HSCs towards different lineage bias (Fig 1E). Remarkably, cluster 1 was present in LPS and PBS conditions, and was driven by expression of megakaryocytic and erythroid lineage genes. Clusters 2 and 3 were dominant in steady‐state conditions (PBS) and almost absent upon LPS treatment. Gene expression defined cluster 2 as a transitional HSC subpopulation exhibiting properties of both cluster 1 and cluster 3, while cluster 3 was mainly defined by expression of lymphoid lineage as well as stem cell genes. Remarkably, cluster 4 and 5 appeared exclusively upon treatment of mice with LPS and were defined by expression of inflammatory genes or myeloid lineage genes, respectively (Fig 1D and E). Interestingly, pseudo‐time analysis, which places cells onto a linear trajectory following a continuum of gene expression changes of cellular states, suggested that upon induction of emergency granulopoiesis, the steady‐state lymphoid‐biased HSC subpopulation (cluster 3) is transcriptionally rewired through an inflammatory intermediate (cluster 4) to a LPS‐specific myeloid‐biased HSC subpopulation (cluster 5; Fig 1F and G and Appendix Table S2). In line with these observations, chromatin accessibility analysis of HSCs isolated from WT mice treated with LPS or PBS control (experimental design as in Fig 1A), corroborated that emergency granulopoiesis at the HSC level was in general marked by opening of inflammatory and myeloid‐biased loci rather than steady‐state lymphoid‐biased loci (Fig 1H and Appendix Table S1). Finally, we confirmed the lineage bias changes at the HSC level by assessing the multipotent progenitor (MPP) distribution, as this is the first population downstream of HSCs where a gating strategy for individual lineage‐biased subpopulations has been reported (Pietras *et al*, 2015). In line with the scRNA‐seq data, we observed a decrease in lymphoid‐biased MPP4 and a substantial expansion of the myeloid‐biased MPP2 and MPP3 subpopulations at 4 h after *in vivo* LPS treatment (Fig 1I and J). Altogether, these results indicate that (i) HSCs are transcriptionally activated at early stages of emergency granulopoiesis, and (ii) that the HSC identity is preferentially changed towards a myeloid‐biased HSC during emergency granulopoiesis.

### Loss of CD201 expression on HSCs is a general event during emergency granulopoiesis and is partially dependent on TLR4/MyD88 signaling axis

**Figure 2:**
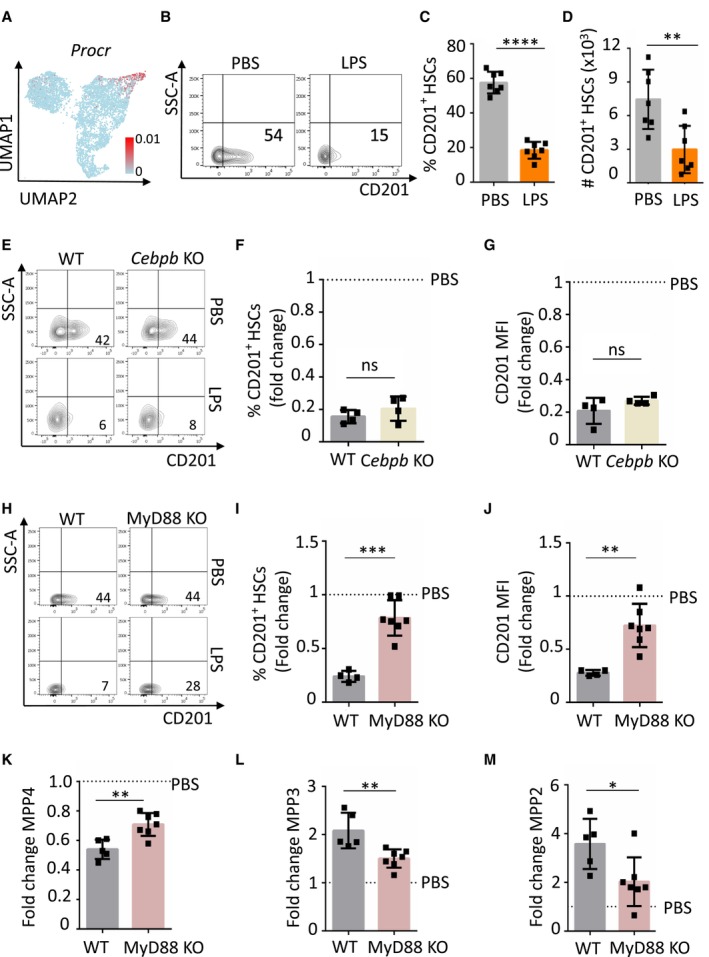
CD201 downregulation during emergency granulopoiesis is partially dependent on TLR4‐MyD88 signaling AUMAP plot color‐coded for the expression of *Procr*.BRepresentative flow cytometry plots of BM cells isolated from mice treated with PBS control or LPS for 4 h. The *x*‐axes indicate CD201 expression. Numbers show percentage of CD201^+^ HSCs.CQuantification of panel b. The *y*‐axis indicates percentage of CD201^+^ HSCs in BM.DAbsolute number of CD201^+^ HSCs in BM isolated from mice treated with PBS control or LPS for 4 h. The *x*‐axis indicates absolute number.ERepresentative flow cytometry plots indicating CD201 expression in HSCs isolated from WT and *Cebpb* KO mice treated with PBS or LPS as indicated for 4 h. Numbers indicate percentage of CD201^+^ HSCs.F, GQuantification of panel (E). Percentage of CD201^+^ HSCs (F) and CD201 mean fluorescence intensity (MFI) (G). *X*‐axes indicate fold change relative to PBS control mice (dashed lines).HRepresentative flow cytometry plots indicating CD201 expression in HSCs isolated from WT and MyD88 KO mice treated with PBS or LPS as indicated for 4 h. Numbers indicate percentage of CD201^+^ HSCs.I, JQuantification of panel (H). Percentage of CD201^+^ HSC (I) and CD201 MFI (J) in WT (gray columns) and MyD88 KO (pink column) mice treated with LPS for 4 h.K–MQuantification of MPP4 (K), MPP3 (L) and MPP2 (M) populations in WT (gray columns) and MyD88 KO (pink column) mice treated with LPS for 4 h. *X*‐axes indicate the fold change to PBS control. Data information: Dashed lines indicate PBS levels. In this figure, data represent mean ± SD from at least two independent experiments. At least four animals were included in each group. Two‐tailed Student's *t*‐test was used to assess statistical significance (*P < 0.05, **P < 0.01, ***P < 0.001, and ****P < 0.0001, ns, not significant). Source data are available online for this figure.

As our scRNA‐seq data suggested that certain HSC clusters are transcriptionally rewired from a lymphoid to a myeloid‐biased during early stages of emergency granulopoiesis, we searched for a cell surface marker that would allow us to track and further characterize this change experimentally. We identified *Procr* gene, coding for the cell surface molecule CD201, as a potential candidate (Fig 1E and Appendix Table S1). In steady‐state conditions, *Procr* levels were high in lymphoid‐biased HSCs, and the expression was diminished upon LPS administration, marking the switch towards a myeloid‐biased HSC subpopulation (Fig 2A). Since *Procr* was one of the top ranking differentially expressed genes (DEG) in the lymphoid‐biased HSC subpopulation (Fig 1E and Appendix Table S1), we carried on to analyze CD201 protein levels on HSCs. Flow cytometric analysis demonstrated that indeed upon LPS administration CD201 protein expression is diminished in HSCs (Fig 2B–D and Appendix Fig S2A). This reduction in CD201 expression was time restricted as CD201^+^ HSC were recovered 24 h upon LPS administration (Appendix Fig S2B). Remarkably, we demonstrated that reduction of CD201 expression in HSCs is a general event during emergency granulopoiesis, as administration of G‐CSF and *Candida albicans* resulted in similar effects (Appendix Fig S3).

Because we observed that CD201 expression marks the lymphoid to myeloid transcriptional switch during emergency granulopoiesis in HSCs, we explored the molecular mechanisms that mediate CD201 downregulation upon LPS administration. Since C/EBPβ is a key transcription factor during emergency granulopoiesis (Hirai *et al*, 2006) and *Cebpb* was one of the top ranking DEG in the LPS specific myeloid‐biased HSCs (Fig 1E and Appendix Table S1), we next investigated whether *Cebpb* deficient mice were able to downregulate CD201 expression upon LPS administration. Therefore, *Cebpb* deficient mice were challenged with LPS and the levels of CD201 were assessed 4 h later. Surprisingly, we observed that *Cebpb* knockout (KO) mice exhibited CD201 downregulation in the HSC compartment upon administration of LPS similarly to WT mice (Fig 2E–G). Since C/EBPβ, mostly induced in an indirect manner during emergency granulopoiesis, showed to be dispensable for CD201 downregulation, we next assessed the contribution of direct pathogen sensing to CD201 downregulation. Because LPS is sensed and signals through the TLR4/MyD88 signaling pathway (Zhang *et al*, 2016), we challenged *Myd88* deficient mice with LPS. Analysis of mice 4 h upon administration demonstrated that MyD88 KO mice were not able to completely downregulate CD201 levels on HSCs (Fig 2H–J), however, a partial reduction was observed when compared to PBS control. In line with these results, we observed that while the lymphoid to myeloid switch was not completely abrogated in these mice, it was significantly reduced compared to WT controls (Fig 2K–M). Altogether, these experiments indicate that downregulation of CD201 in HSCs is a universal event during emergency granulopoiesis and that, to a certain extent, it is dependent on the TLR4/MyD88 signaling axis, while C/EBPβ is dispensable.

### CD201 ^−^ and CD201 ^+^ HSCs exhibit distinct lineage differentiation output

**Figure 3:**
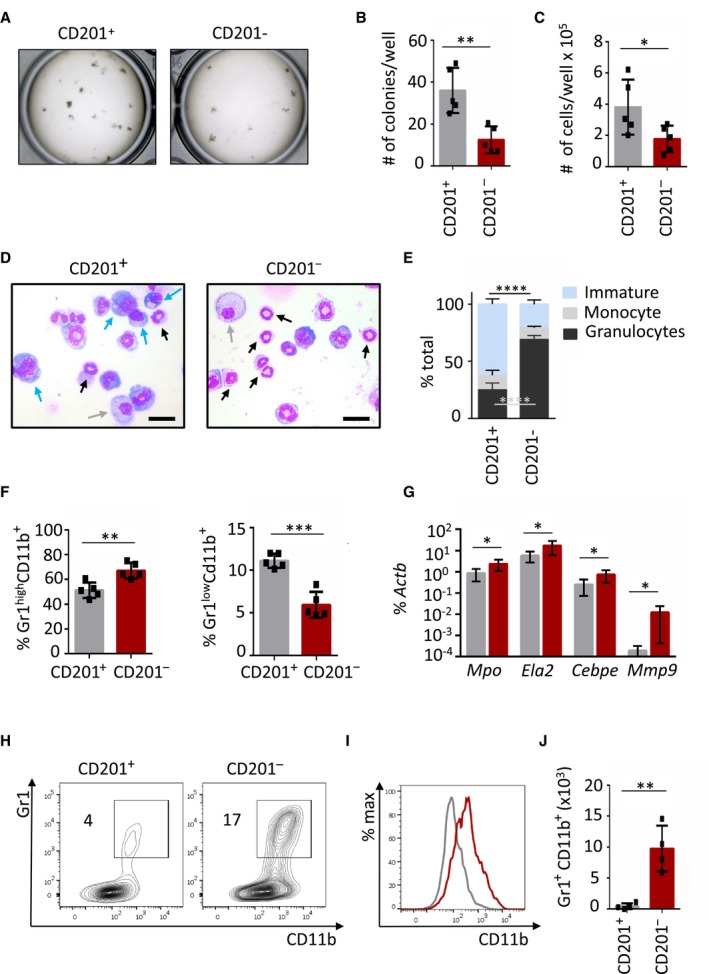
CD201^−^ and CD201^+^ HSCs lineage differentiation output in culture Macroscopic pictures of colony culture assays using MethoCult GF M3434 of CD201^+^ and CD201^−^ HSCs isolated from WT mice. A total of 100 HSCs was plated per well. Cultures were analyzed at day 7 of culture.Quantification of panel (A). *Y*‐axis indicates the absolute number of colonies per well.Absolute cell counts per well.Representative pictures of cells cytospun from colony culture assays. Cytospins were stained with May‐Grünwald Giemsa. Scale bar represents 20 μm. Blue arrows point at immature cells (medium to large cells with big nucleus and scant and dark‐blue cytoplasm), gray arrows at monocytes (large and round cells with round nucleus and light‐blue cytoplasm), and black arrows at granulocytes (smaller cells characterized by a ring shape or lobulated nucleus).Differential cell counting based on cell morphology. 200–300 cells per cytospin were assessed. *Y*‐axis indicates the percentage of immature cells (blue bars), monocytes (gray bars), and granulocytes (black bars).Flow cytometric analysis of cells harvested from semi‐solid cultures. *Y*‐axes indicate percentage of mature granulocytes (left) and immature granulocytes (right).Quantitative RT–PCR from cells harvested from CD201^+^ and CD201^−^ cultures. Expression of *Mpo*, *Ela2*, *Cebpe*, and *Mpp9* is indicated. The *y*‐axis represents relative expression compared to *Actb* control. Each group includes values for six independent cultures.Representative flow cytometric plots of CD201^+^ (left) and CD201^−^ (right) liquid cultures. *Y*‐axes indicate Gr1 expression and *x*‐axes CD11b expression. Numbers indicate percentage of Gr1^+^ CD11b^+^ cells.Representative histogram plot of CD201^+^ (gray line) and CD201^−^ (red line) cultures. *X*‐axis indicates CD11b expression.Absolute number of Gr1^+^ CD11b^+^ mature granulocytic counts in liquid cultures. Data information: In this figure, data represent mean ± SD from two independent experiments. Each symbol represents values for one mouse. two‐tailed Student's *t‐*test was used to assess statistical significance (*P < 0.05, **P < 0.01 and ***P < 0.001). Source data are available online for this figure.

Because our results suggested that HSCs can be divided based on CD201 expression, we investigated the differentiation potential of CD201^−^ and CD201^+^ HSCs in culture. When subjected to myeloid culture conditions, CD201^−^ HSCs formed fewer colonies, smaller in size and with reduced cell numbers, and mostly gave rise to mature granulocytes in comparison to CD201^+^ HSCs, as demonstrated by analysis of the colonies (Fig 3A–C), cell morphology (Fig 3D and E), flow cytometric analysis (Fig 3F), and RT–PCR (Fig 3G). The preferential granulocytic output of CD201^−^ HSCs over CD201^+^ HSCs was also demonstrated in liquid cultures (Fig 3H–J).

**Figure 4:**
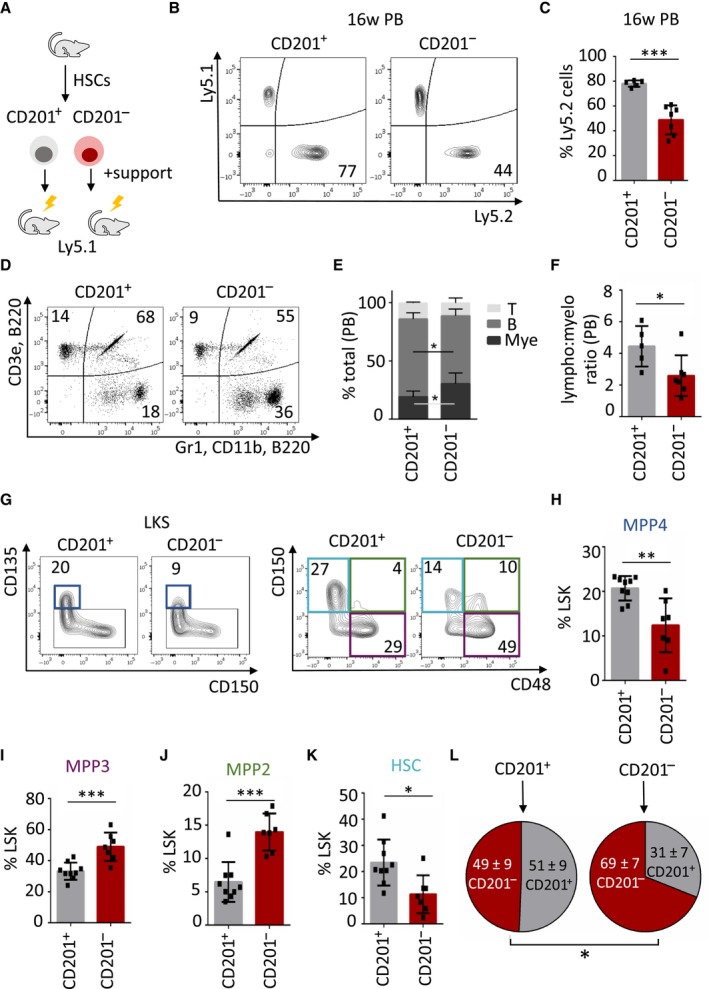
CD201^−^ and CD201^+^ HSCs lineage differentiation output upon transplantation AGraphical representation of the experimental design. 700 CD201^+^ or CD201^−^ HSCs were sorted from Ly5.2 mice and transplanted into lethally irradiated Ly5.1 recipients together with 0.5 × 10^6^ Ly5.1 support BM.BRepresentative flow cytometric analysis of peripheral blood from recipient mice 16 weeks after transplantation. Left panel represents a recipient mouse that received CD201^+^ HSCs, right panel CD201^−^ HSCs. *Y*‐axes indicate percentage of Ly5.1‐derived BM support cells and *y*‐axes percentage of Ly5.2‐derived donor HSCs. Numbers indicate percentage of Ly5.2^+^ cells.CQuantification of panel (B). The *y*‐axis indicates percentage of Ly5.2^+^ cells. At least five animals were included in each group.DTri‐lineage reconstitution in peripheral blood 16 weeks upon transplantation. Upper left panel indicates percentage of donor‐derived Ly5.2^+^ T cells, upper right panel indicates percentage of donor‐derived Ly5.2^+^ B cells, and lower right panel indicates percentage of donor‐derived Ly5.2^+^ myeloid cells. Recipient mice received CD201^+^ (left) or CD201^−^ (right) HSCs.EQuantification of flow cytometric data of panel (D). *Y*‐axis indicates the percentage of donor‐derived T cells, B cells, and myeloid cells.FAbundance of lymphoid versus myeloid cells. *Y*‐axis indicates the lymphoid to myeloid ratio in peripheral blood derived from CD201^+^ or CD201^−^ HSCs.GFlow cytometric strategy to determine CD201^+^ and CD201^−^ HSC‐derived progenitor and stem cells. Numbers indicate percentages of LKS. Dark blue boxes indicate multipotent progenitor 4 (MPP4), violet boxes indicate MPP3, green boxes indicate MPP2, and light blue boxes indicate HSCs.H–KQuantification of panel (G). *Y*‐axes indicate percentages in LKS population. At least six animals were included in each group.LAnalysis of the BM HSC compartment isolated from mice transplanted with CD201^+^ (left) or CD201^−^ (right) HSCs. Circles indicate percentage of CD201^+^ and CD201^−^ HSCs upon 16 weeks of transplantation. Data information: Data represent mean ± SD from two independent experiments. Two‐tailed Student's *t*‐test was used to assess statistical significance (*P < 0.05, **P < 0.01, ***P < 0.001). Source data are available online for this figure.

Next, we investigated the preferential CD201^−^ and CD201^+^ HSC lineage output *in vivo*. Thus, CD201^−^ and CD201^+^ HSCs (Ly5.2) were sorted and transplanted into lethally irradiated congenic mice (Ly5.1) in the presence of BM support (Ly5.1) (Fig 4A). Blood and BM of recipient mice were analyzed 16 weeks after transplantation and the percentage of Ly5.2‐derived cells was determined. We observed that CD201^+^ HSCs engrafted better than CD201^−^ HSCs (Fig 4B and C, and Appendix Fig S4A). Accordingly, the number of mice reconstituted with CD201^+^ HSCs was significantly higher than the number of mice reconstituted with CD201^−^ HSCs (Appendix Fig S4B). In terms of lineage reconstitution, we determined that CD201^+^ HSCs exhibited a higher lymphoid output than CD201^−^ HSCs, which on the contrary had an enhanced myeloid production (Fig 4D–F and Appendix Fig S4C–E). Interestingly, the distinct lineage bias could be already detected at the level of progenitor cells, where we observed that CD201^+^ HSCs favored MPP4 production while CD201^−^ HSCs gave rise to significantly more MPP3 as well as MPP2 subpopulations (Fig 4G–J). Interestingly, we also noticed that HSC reconstitution was more efficient from CD201^+^ HSCs than from CD201^−^ HSCs (Fig 4K). Nevertheless, we observed that upon transplantation and hematopoietic recovery both HSC subpopulations gave rise to CD201^−^ and CD201^+^ HSCs, although retaining certain degree of fidelity to the donor population (Fig 4L). These results indicate that in resting conditions following hematopoietic transplantation, HSCs can be divided according to CD201 expression, allowing the identification and isolation of myeloid‐biased (CD201^−^) or lymphoid‐biased (CD201^+^) HSCs. Since CD150 has been previously defined as a marker to distinguish myeloid‐ from lymphoid‐biased HSCs, we combined and compared CD150 and CD201 expression in HSCs, but could not correlate the combination of these two markers to a unique HSC lineage bias (Appendix Fig S4F). Altogether, these results suggest that CD201 expression on HSCs reveals a preferential lymphoid‐ versus myeloid‐bias in steady‐state conditions, and that under emergency granulopoiesis CD201 marks the lymphoid to myeloid transcriptional switch in HSCs.

### CD201 expression on HSCs functionally contributes to emergency granulopoiesis

**Figure 5:**
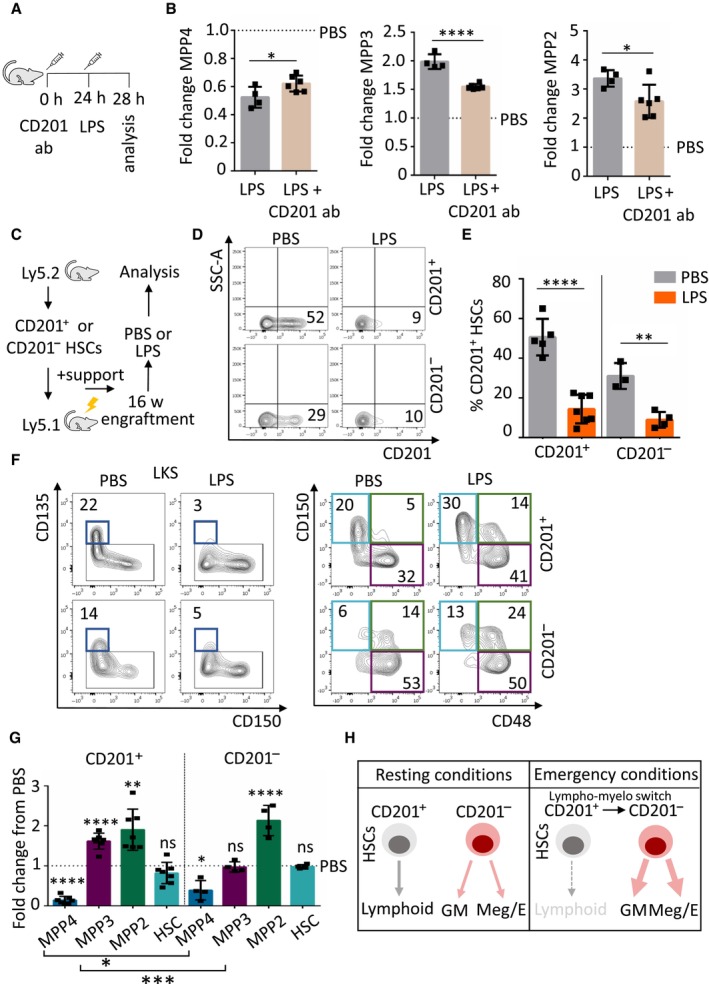
Role of CD201 in the lineage switch and contribution of CD201^+^ and CD201^−^ HSCs to emergency granulopoiesis Schematic representation of the experimental design. Mice were treated at the indicated time points with CD201 function‐blocking antibodies (CD201 ab) and LPS (35 μg).Quantification of MPP4, MPP3, and MPP2 populations in mice treated with LPS control (gray columns) or CD201‐function blocking antibodies followed by LPS administration (brown columns). *X*‐axes indicate the fold change to PBS control. Dashed lines indicate PBS levels.Graphical representation of the experimental design. CD201^+^ and CD201^−^ chimeras were challenged with LPS or PBS control 16 weeks after transplantation. Mice were sacrificed and analyzed 4 h after the challenge.Flow cytometric analysis of BM from recipient mice that received CD201^+^ (top) or CD201^−^ (bottom) HSCs and were challenged with PBS (left) or LPS (right). *X*‐axes indicate CD201 expression in donor‐derived (Ly5.2^+^) HSCs. Numbers indicate percentages.Quantification of panel (D). *Y*‐axis indicates percentage of donor‐derived Ly5.2^+^ CD201^+^ HSCs in chimeras that received CD201^+^ or CD201^−^ HSCs. Analysis was done 4 h after treatment. At least three animals were included in each group.Flow cytometric analysis of BM from recipient mice that received CD201^+^ (top) or CD201^−^ (bottom) HSCs and were challenge with PBS (left) or LPS (right). Flow cytometry plots indicate gating strategy to identify progenitor and stem cell populations. Dark blue boxes indicate multipotent progenitor 4 (MPP4), violet boxes indicate MPP3, green boxes indicate MPP2, and light blue boxes indicate HSCs. Numbers indicate percentages of LKS. Analysis was done 4 h after treatment.Quantification of panel (F). *Y*‐axis indicates fold change from PBS‐treated mice to LPS‐treated mice. Dashed line indicates PBS levels. At least six animals were included in each group.Graphical summary. Left panel indicates HSC behavior in resting conditions and right panel indicates HSC behavior during emergency granulopoiesis. HSCs could be divided according to CD201 expression. In steady‐state, CD201^+^ HSCs contribute to lymphoid production, while CD201^−^ HSCs mainly supply granulocytic/monocytic (GM) and megakaryocytic/erythroid (Meg/E) demands. Under emergency granulopoiesis, CD201^+^ HSCs are transcriptionally rewired and CD201 expression is diminished, contributing to the enhanced GM and Meg/E production at the expenses of the lymphoid lineage supply. Data information: Data represent mean ± SD from two independent experiments. Two‐tailed Student's *t*‐test was used to assess statistical significance (*P < 0.05, **P < 0.01, ***P < 0.001, ****P < 0.0001, not significant). Source data are available online for this figure.

Next, we investigated whether CD201 actively contributes to emergency granulopoiesis or whether it merely serves as a marker to trace the lineage switch. Mice were treated with a CD201 function‐blocking antibody or PBS control as previously described (Magisetty *et al*, 2020), followed 24 h later by an LPS injection. Four hours upon LPS administration, mice were sacrificed and emergency granulopoiesis at the stem and progenitor level was assessed (Fig 5A). We observed that upon LPS stimulation mice that received one injection of CD201 blocking antibody exhibited hallmarks of emergency granulopoiesis, including reduction of the MPP4 population and expansion of the MPP3 and MPP2 compartments. However, the response was significantly lower than in non‐blocking antibody treated control mice (Fig 5B). Altogether, these results suggest that CD201, in addition to marking the lymphoid to myeloid switch during emergency granulopoiesis, functionally contributes to the process although its blockage is not sufficient to completely abolish the response to LPS.

### CD201 ^+^ and CD201 ^−^ HSCs contribute in a different manner to emergency granulopoiesis

To further explore the individual contribution of CD201^+^ and CD201^−^ HSCs to emergency granulopoiesis, we next investigated the response of these cells to LPS challenge. CD201^+^ or CD201^−^ HSCs were transplanted into lethally irradiated mice, and 16 weeks later, emergency granulopoiesis was induced by LPS (Fig 5C). In line with our results in Fig 2, we observed a major decline in the percentage of CD201^+^ HSCs 4 h after inducing emergency granulopoiesis (Fig 5D and E). Next, recipient mice were analyzed to assess the changes in the BM progenitor populations. Interestingly, we observed a reduction of lymphoid‐biased MPP4 in both cases, with a more pronounced reduction in the mice transplanted with CD201^+^ HSCs, and an increase of myeloid‐biased MPP2 population (Fig 5F and G). Interestingly, the expansion of myeloid‐bias MPP3 subpopulation was not observed when chimeras transplanted with CD201^−^ HSCs were challenged with LPS, since in PBS conditions these cells are already myeloid‐biased. Nevertheless, we detected a prominent and significant expansion of the myeloid‐bias MPP3 subpopulation in chimeras transplanted with CD201^+^ HSCs upon LPS administration (Fig 5F and G). Altogether, these experiments support our scRNA‐seq data, and demonstrate that during emergency granulopoiesis two different subsets of HSCs respond in a different manner to the increased granulocytic demands and compromise the lymphoid output (Fig 5H).

### Different regulatory mechanisms sense and mediate emergency granulopoiesis in CD201 ^−^ and CD201 ^+^ HSCs

**Figure 6:**
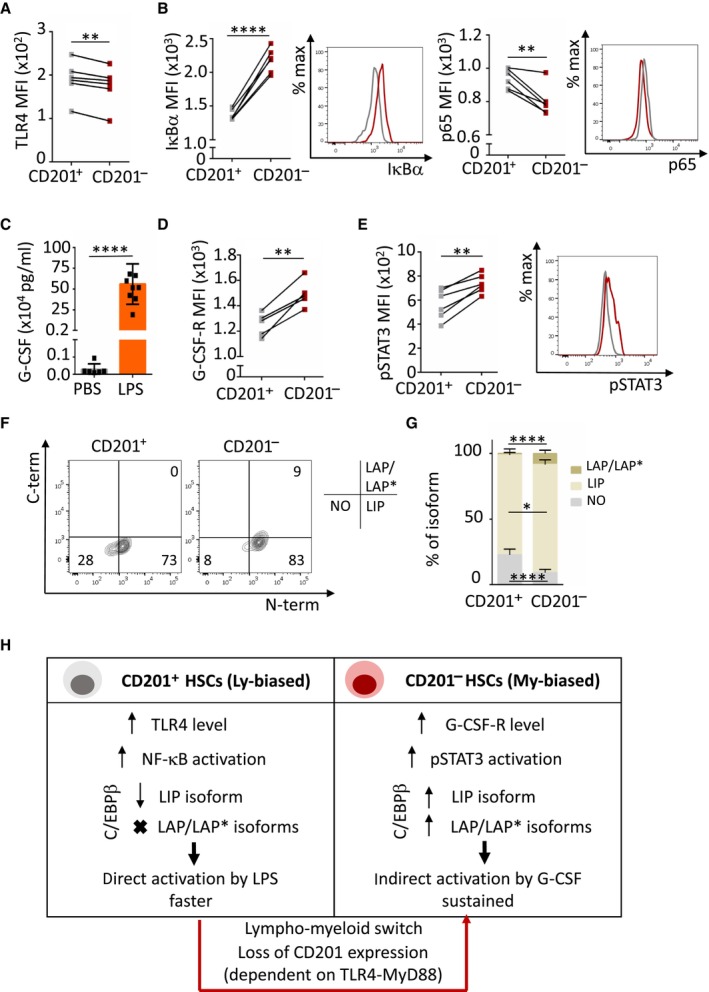
Two different molecular mechanisms regulate emergency granulopoiesis in CD201^−^ and CD201^+^ HSCs Quantification of TLR4 mean fluorescence intensity (MFI) in CD201^+^ (gray) and CD201^−^ (red) HSCs.Quantification and representative histogram plot of IκΒα (left) and p65 (right) MFI in CD201^+^ (gray) and CD201^−^ (red) HSCs upon 5 min of LPS stimulation in culture (*n* = 6 samples per condition).G‐CSF quantification in serum of mice treated with LPS or PBS control for 4 h. *X*‐axis indicates G‐CSF levels.Quantification of G‐CSF‐receptor (G‐CSF‐R) MFI in CD201^+^ (gray) and CD201^−^ (red) HSCs.Quantification (left) and representative histogram plot (right) of pSTAT3 MFI in CD201^+^ (gray) and CD201^−^ (red) HSCs upon 5 min of G‐CSF stimulation in culture (*n* = 6 samples per condition).Representative flow cytometry contour plots showing levels of the distinct C/EBPβ isoforms in CD201^+^ and CD201^−^ HSCs isolated from WT mice. Numbers indicate percentage of negative cells (lower left quadrant), C/EBPβ LIP isoform expressing cells (lower right quadrant), and C/EBPβ LAP/LAP* isoforms expressing cells (upper right quadrant).Quantification of the panel (F). *X*‐axis indicates the percentage of the distinct C/EBPβ isoforms (*n* = 6 samples per condition).Illustration summarizing the two distinct molecular mechanisms mediating emergency granulopoiesis in CD201^+^ and CD201^−^ HSCs. Data information: Data represent mean ± SD from two independent experiments. In panels (A), (C), and (D) each datapoint represents 1 mouse. In panels (B), (E), (F), and (G) each datapoint represents a pool of sorted CD201^+^ or CD201^−^ HSCs from two mice. Two‐tailed Student's *t*‐test was used to assess statistical significance (*P < 0.05, **P < 0.01, ****P < 0.0001). Source data are available online for this figure.

Given the distinct functional behavior of CD201^−^ and CD201^+^ HSCs during emergency granulopoiesis and the lack of CD201 downregulation upon LPS treatment in MyD88 KO mice, we next investigated whether these two distinct HSC subsets preferentially sense the infection and initiate the response in a different manner. We observed that CD201^+^ HSCs express higher levels of TLR4 than CD201^−^ HSCs and accordingly, CD201^+^ HSCs have higher activation of NF‐κB signaling upon *in vitro* LPS stimulation (Fig 6A and B). Second, we determined that G‐CSF levels were highly upregulated in serum of mice challenged with LPS 4 h upon injection (Fig 6C), suggesting that indirect sensing could also participate in the activation of emergency granulopoiesis at the HSC level. Remarkably, we observed that CD201^−^ HSCs express higher levels of G‐CSF‐R than CD201^+^ HSCs and accordingly, CD201^−^ HSCs have a higher activation of pSTAT3 signaling upon *in vitro* G‐CSF‐stimulation (Fig 6D and E). Next, since we previously reported that distinct C/EBPβ isoforms sequentially regulate hematopoietic stem and progenitor regeneration upon stress (Sato *et al*, 2020), we assessed the abundance of these isoforms in CD201^+^ and CD201^−^ HSCs. Interestingly, we determined that while the LIP isoform of C/EBPβ, important for cell proliferation, is present in both CD201^+^ and CD201^−^ HSCs, the LAP/LAP* isoforms, important for myeloid differentiation, are present only in the CD201^−^ HSCs fraction (Fig 6F and G). Altogether, these results suggest that emergency granulopoiesis is supported by two individual populations of HSCs which employ distinct mechanisms to sense and to respond to acute infections (Fig 6H). On one hand, the lymphoid‐bias CD201^+^ HSCs represent a first response, facilitated by a direct sensing of LPS by TLR4 and activation of downstream NF‐κΒ signaling. On the other hand, the myeloid‐bias CD201^−^ HSCs respond in an indirect manner to G‐CSF, increasing the amount of pSTAT3, and upregulating the levels of LAP/LAP* C/EBPβ isoforms. Thus, the switch from CD201^+^ to CD201^−^ HSCs facilitates both fast and sustained emergency granulopoiesis by employing distinct molecular pathways.

## Discussion

Precise and efficient immune response is essential to eliminate pathogens and sustain health. While immune cells eliminate and remove the pathogens, the response is usually initiated in rather immature populations which orchestrate the precise adjustment of the hematopoietic needs to supply sufficient amount of mature immune cells (Hirai *et al*, 2006; Manz & Boettcher, 2014). Emergency granulopoiesis is traditionally understood as a process mediated by myeloid progenitors. In the present study, we show that emergency granulopoiesis is initiated already at the level of the most immature HSCs shortly after LPS treatment. This is in line with previously published data showing that HSPCs respond to inflammatory stimuli *in vitro* early after stimulation (Mann *et al*, 2018). Accordingly, previous studies have shown that under emergency conditions, hematopoiesis is supported by HSCs rather than MPPs (Haas *et al*, 2015; Singh *et al*, 2018). However, we cannot exclude that during emergency granulopoiesis the MPPs are further supporting the increased granulocytic demands.

The favoring of granulopoiesis over lymphopoiesis is a general phenomenon in emergency granulopoiesis, as B lymphopoiesis is inhibited at the progenitor level by the increased levels of G‐CSF during emergency granulopoiesis (Day *et al*, 2015). Accordingly, Pietras and colleagues demonstrated that transcriptional reprogramming inhibiting lymphopoiesis occurs in distinct multipotent progenitor subpopulations to support hematopoietic regeneration (Pietras *et al*, 2015). Remarkably, our results demonstrate that the inhibition of lymphopoiesis occurs much earlier during emergency granulopoiesis, already at the HSC level. The HSC compartment is comprised of different lineage‐biased HSCs (Hirschi *et al*, 2017). Here, we identified a steady‐state lymphoid‐bias HSC subpopulation, which upon the initiation of emergency granulopoiesis gains a pro‐inflammatory gene signature and is transcriptionally rewired to a myeloid‐bias HSC pool. We provide evidence that the HSC lineage‐bias can be reverted under specific hematopoietic needs, pointing at the central role of the flexibility of the HSC pool. Nevertheless, it is surprising that under steady state conditions, we observed a lack of a myeloid‐biased HSCs cluster. Yet, using scRNAseq it was previously reported that in human BM a clear separation into single lineages was only observed among progenitors and not HSCs (Velten *et al*, 2017), allowing us to speculate that similarly, in murine BM not all cell identities are clearly marked and individually separated at the HSC level. Further, we identified two distinct populations (#1 and #2) that transcriptionally represent a megakaryocyte‐erythroid HSC lineage bias. Since megakaryocyte/erythroid‐biased multipotent progenitors are able to produce myeloid cells in steady‐state (Pietras *et al*, 2015), we can speculate that a similar cell fate can take place in HSCs under PBS conditions.

CD201, also known by endothelial protein C receptor (EPCR), is a type I transmembrane glycoprotein in the CD1/major histocompatibility complex family and is encoded by *PROCR* gene (Fukudome & Esmon, 1994; Rothbarth *et al*, 1999). Originally, CD201 was found to be expressed on endothelial cells and to play a major role in the blood coagulation pathway (Stearns‐Kurosawa *et al*, 1996; Mohan Rao *et al*, 2014). More recently, CD201 has been also found to be highly expressed on HSCs, however, its function on HSCs remains rather elusive (Balazs *et al*, 2006). Here, we determined that CD201 marks and actively participates in the lymphoid to myeloid transcriptional switch on HSCs under emergency granulopoiesis. Despite the identification and characterization of the murine *Epcr* promoter, which controls constitutive CD201 expression (Simmonds & Lane, 1999; Gu *et al*, 2000), the mechanisms that mediate CD201 downregulation in emergency granulopoiesis remained unknown. We determined that the transcription factor CEBPβ was dispensable for CD201 downregulation in HSCs, while we observed a partial contribution of the MyD88 adaptor protein. However, other factors that further mediate CD201 downregulation during emergency granulopoiesis are yet to be determined. As the inflammatory TLR4 signaling is carried out by the precise cooperation of the MyD88 and TRIF adaptors, and their respective downstream signaling pathways (Zhang *et al*, 2016), and TRIF has been shown to be the major signaling adaptor for TLR4 on HSCs (Takizawa *et al*, 2017), the contribution of TRIF in combination with MyD88 should be further investigated to fully understand the mechanisms driving CD201 downregulation on HSCs during emergency granulopoiesis.

A frequent limitation in stem cell biology is the restricted number of unique cell surface markers available allowing us to sort and isolate distinct HSC subpopulations. CD201 is a marker currently used to enrich for “true” HSCs in murine BM, and has been reported to also mark long‐term HSCs in expanded human cord blood (Balazs *et al*, 2006; Kent *et al*, 2009; Vazquez *et al*, 2015; Papa *et al*, 2020). In line with these reports, in our settings CD201^+^ HSCs show enhanced colony‐forming potential in culture, as well as high engraftment and increased HSC reconstitution *in vivo*. Nevertheless, we observed that CD201 expression on HSCs is linked to a lymphoid‐bias output in steady‐state conditions, questioning the use of CD201 as a “true” HSC marker. Further, transplantation of CD201^+^ HSCs gave rise to the reconstitution of both CD201^+^ and CD201^−^ HSCs, and similarly transplantation of CD201^−^ HSCs was also able to generate CD201^+^ and CD201^−^ HSCs, suggesting a full hematopoietic potential in both types of HSCs. Moreover, here we report that upon induction of emergency granulopoiesis CD201 expression is lost, marking the lympho‐myeloid switch in HSCs. Altogether, these findings suggest that CD201 as an HSC marker should be used with caution, as the expression is not stable in inflammatory conditions. In line with our observations, it was previously reported that the HSC compartment could be divided based on the expression of CD201 and CD34 into a continuum of cells with different lineage priming and activity under chronic inflammatory conditions (Rabe *et al*, 2020). Moreover, they also showed a reduction in frequency of the CD201^+^ CD34^−^ compartment upon chronic IL‐1 treatment, which together with our results using G‐CSF and *C. albicans* suggests that CD201 downregulation might be a universal phenomenon under different types of inflammatory stress. Further, based on previous publications (Beerman *et al*, 2010) and our observations, one would expect that expression of CD150 and CD201 are inversely correlated, which would support either their lymphoid or myeloid bias. However, our data revealed a more complex scenario: we observed that CD150^−^ HSCs can be sub‐divided in CD201^−^ and CD201^+^ HSCs, and similarly, CD150^+^ HSCs can be sub‐divided in CD201^−^ and CD201^+^ HSCs. It would be interesting to sort these four distinct populations and perform transplantation assays, or *in vivo* lineage tracing experiments, to identify their fate and lineage bias.

Recent reports point at the contribution of HSCs to emergency granulopoiesis, although understanding how, remained rather elusive. Here, we provide evidence demonstrating that emergency granulopoiesis is supported by a subpopulation of HSCs which upon pathogen sensing undergoes a radical transcriptional rewiring that promotes their myeloid output. Initially, the pathogen is directly sensed by TLR4 on the surface of a steady state lymphoid‐biased CD201^+^ HSCs, causing a rapid activation of the downstream NF‐κB signaling pathway. Subsequently, the lymphoid‐myeloid transcriptional switch, marked by the loss of CD201 expression occurs and emergency granulopoiesis is then supported by myeloid‐biased CD201^−^ HSCs. CD201^−^ HSCs respond to the infection in an indirect manner through G‐CSF‐R on their surface and exhibit enhanced pSTAT3 activation and elevated LAP/LAP* C/EBPβ isoforms, cellular mechanisms known to promote myeloid differentiation and granulocytic production. These observations expand our understanding on emergency granulopoiesis, which so far was understood as a process in which TLR4 expression in hematopoietic cells was dispensable (Boettcher *et al*, 2012, 2014). In brief, we propose that the switch from CD201^+^ to CD201^−^ HSCs facilitates both fast and sustained emergency granulopoiesis by employing two distinct mechanisms. We hypothesize that this dual regulatory mechanism ensures the supply of sufficient granulocytes to eliminate the infection while it preserves an HSC subpopulation that re‐establishes the lymphoid production once the infection has been cleared. We believe that further studies are needed to better understand the mechanisms which secure lymphoid lineage production during and after emergency granulopoiesis. Ultimately, we provide insights into the cellular, transcriptional, and mechanistic properties that determine HSC fate during emergency granulopoiesis. These observations highlight the possibility that in the context of inefficient immune responses, for instance in patients suffering from neutropenia, targeted stimulation of a subset of HSCs could boost the granulocytic supply.

## Material and Methods

### Animal models

Eight‐ to 15‐week‐old WT C57BL/6 mice, straight whole‐body *Cebpb* deficient mice (Screpanti *et al*, 1995), and straight whole‐body *Myd88* deficient mice (Adachi *et al*, 1998) were employed for this study. For the transplantation assays, congenic strains Ly5.1 and Ly5.2 were used. Mice were maintained under specific pathogen free conditions in the animal facility of the Institute of Molecular Genetics of the CAS, except for *Cebpb* deficient mice which were maintained in the specific pathogen‐free facility at Tokyo University of Pharmacy and Life Sciences. All experiments were approved by the ethical committee of the respective institutes (approval numbers: AVCR 7141‐2022 SOV II, AVCR 5636‐2023 SOV II, and L23‐14).

### Induction of emergency granulopoiesis

To study early time points of emergency granulopoiesis, mice received a single intraperitoneal injection of 35 μg of ultrapure LPS from *Escherichia coli* 0111:B4 (InvivoGen, San Diego, CA, USA). Analysis was done 4 h after the challenge, unless otherwise indicated. Alternatively, 250 μg/kg of body weight of human recombinant G‐CSF (Neupogen, Amgen, Thousand Oaks, CA, USA) was administered intraperitoneally and the mice were analyzed 4 h later. Infection with *C. albicans* was performed as described previously with minor modifications (Kardosova *et al*, 2018). Briefly, *C. albicans* (18804, American Type Culture Collection) was plated on Sabouraud dextrose agar plates and grown for 24 h at 37°C. Plates were stored at 4°C for a maximum of 4 weeks. Before each experiment, one colony was picked from the plate and grown in 5 ml Sabouraud dextrose broth (Sigma‐Aldrich, St. Louis, MO, USA) at 37°C overnight. Fungi were washed twice with sterile PBS and resuspended in PBS. For induction of disseminated candidiasis, mice were injected intravenously in the right tail vein with 1 × 10^6^ colony forming units (CFUs)/25 g body weight. The CFU dose counts were verified by diluting and plating the same suspensions with which animals were inoculated. Mice were sacrificed and analyzed 24 h after the injection.

### Administration of CD201 function‐blocking antibody *in vivo*

CD201 blocking antibody (16‐2012‐83, Invitrogen) was administered as previously described (Magisetty *et al*, 2020). Briefly, mice were injected intraperitoneally with a single dose CD201 blocking antibody (1 mg/kg body weight) 24 h before induction of emergency granulopoiesis by LPS. Mice were sacrificed and analyzed 4 h after LPS or PBS (control) injection.

### Flow cytometric analysis and HSC sorting

WT mice were sacrificed by cervical dislocation, femurs and tibias were isolated, and crunched using pestle and mortar. After obtaining single‐cell suspensions, red blood cells were lysed. Cells were then labeled with fluorescence‐conjugated antibodies and analyzed on Symphony instrument (BD Biosciences, San Jose, CA, USA). Antibodies used for phenotypic analysis were: Gr1 PE (RB6‐8C5), CD11b APC (M1/70), anti‐mouse Lineage Cocktail Pacific Blue (including CD3 [17A2]; Gr1 [RB6‐8C5]; CD11b [M1/70]; CD45R/B220 [RA3‐6B2]; TER‐119 [Ter‐119]), c‐Kit PE or APC (2B8), Sca‐1 APC or BV605 (D7), CD48 FITC (HM48‐1), CD150 Pe‐Cy7 (TC15‐12F12.2), CD201 PE (RCR‐16). For the cell surface staining of TLR‐4 and G‐CSF‐R, the cells were first stained with cell surface markers to distinguish CD201^+^ or CD201^−^ HSCs and then fixed with formaldehyde for 30 min to prevent receptor internalization. The fixed samples were stained with either TLR4 APC (MTS510) or G‐CSF‐R Alexa Fluor® 488 (Cys26‐Asp626). Transplanted mice were analyzed using Ly5.1 FITC, PE‐CF597, or PE‐Cy7 (A20), Ly5.2 PE‐Cy7 or BUV737 (104), Gr1 APC or PE (RB6‐8C5), CD11b APC or FITC (M1/70), CD45/B220 APC or PE (RA3‐6B2), CD3ε PE (145‐2C11), c‐Kit PE (2B8), Sca‐1 BV605 (D7), CD48 FITC (HM48‐1), CD150 PE‐Cy7 (TC15‐12F12.2), CD135 APC (A2F10), CD201 PE (RCR‐16). Preparation of BM HSCs for sorting and transplantation was a two‐step process. First, the Lin^+^ fraction of the BM cells was labeled using biotinylated lineage markers: CD45/B220 (RA3‐6B2), CD3 (145‐2c11), Ter119 (TER‐119), Gr1 (RB6‐8C5), and CD11b (M1/70). These cells were further labeled with anti‐biotin magnetic beads (Miltenyi Biotec, Bergisch Gladbach, Germany) and depleted on a MACS separator (Miltenyi Biotec, Bergisch Gladbach, Germany) according to the manufacturer's protocol. Second, the Lin^−^ fraction of the BM was labeled with the following antibodies: c‐Kit PE, c‐Kit APC (2B8), Sca‐1 APC, Sca‐1 BV605 (E13‐161.7), CD48 FITC (HM48‐1), CD150 Pe‐Cy7 (TC15‐12F12.2), CD201 PE (RCR‐16), and streptavidin‐eFluor450. Influx instrument (BD Biosciences, San Jose, CA, USA) was employed to sort HSCs according to the following sorting strategy (Danek *et al*, 2020): Lin^−^, c‐Kit^+^, Sca‐1^+^, CD48^−^ CD150^+^. In all flow cytometric sorting and analyses, Hoechst 33258 was added to cell suspensions to exclude dead cells. Antibodies were purchased from BD Biosciences (San Jose, CA, USA), eBioscience (San Diego, CA, USA), Bio‐Techne or BioLegend (San Diego, CA, USA). Data were obtained using Diva software (BD Biosciences, San Jose, CA, USA) and analyzed using FlowJo software (Tree Star Incorporation, Ashland, OR, USA).

### Flow cytometry analysis of transcriptional factors

For the analysis of pSTAT3, IκB alpha, p65 and C/EBPβ isoforms, CD201^+^ or CD201^−^ HSCs were pooled from 2 WT mice for each sample. The cells were sorted into 150 μl of StemSpan SFEM (Stemcell Technologies, Vancouver, BC, Canada) and stimulated for 5 min at 37°C with either 100 ng/ml LPS or 100 ng/ml G‐CSF (Neupogen, Amgen, Thousand Oaks, CA, USA). Cells were then fixed and permeabilized using the Transcription Factor Buffer Set (BD Biosciences, Franklin Lakes, NJ). Cells stimulated with G‐CSF were stained with FITC anti‐STAT3 (Tyr7054) antibody from BioLegend (San Diego, CA, USA) overnight. Cells stimulated with LPS were stained with IκB alpha antibody conjugated with Alexa Fluor 488 (L35A5, Cell Signaling Technology) or NF‐κB p65 antibody (L8F6, Cell Signaling Technology) overnight. Cells stained with p65 antibody were stained the next day with a respective secondary antibody conjugated with Alexa Fluor 488.

C/EBPβ isoforms were analyzed as described previously with minor modifications (Sato *et al*, 2020). After fixation, cells were first stained with C‐terminal antibody (H‐7, sc‐7962, Santa Cruz Biotechnology) and N‐terminal antibody (LAP, 3087, Cell Signaling Technology) and then with respective secondary antibodies conjugated with PerCP/Cy5.5 (goat anti mouse) and FITC (donkey anti rabbit) BioLegend (San Diego, CA, USA).

### Single cell RNA sequencing (scRNA‐seq)

Ten C57BL/6 mice (10–12 weeks old) were injected with LPS as described above and sacrificed 4 h after the injection. Additional 10 C57BL/6 mice were injected with PBS as a control and sacrificed 4 h later. Blood and BM cells were isolated and processed, and granulocytic response was assessed by cell counts and FACS analysis. HSCs (Lin^−^ c‐Kit^+^ Sca1^+^ CD48^−^ CD150^+^) were sorted as described above. Next, 20,000 cells were pooled for each sample and viability was assessed by trypan blue in TC20 cell counter. Single cell RNA sequencing libraries were prepared using Chromium controller instrument and Chromium next gem single‐cell 3′ reagent kit (version 3.1) according to the manufacturer's protocol (both 10× Genomics, Pleasanton, CA, USA) targeting at 4,000 cells per sample. The quality and quantity of the resulting cDNA libraries was determined using Agilent 2100 Bioanalyzer (Agilent, Santa Clara, CA, USA). The libraries were sequenced separately in two runs of NextSeq 500 sequencer using NextSeq 500/550 high output kit (both Illumina, San Diego, CA, USA) according to the manufacturer's protocol with read length of 54 cycles for cDNA fragment.

### scRNA‐seq analysis

10× Cellranger software (version 4.0.0) was used for demultiplexing, merging of data from multiple runs and final count matrices generation with GRCm38 genome (Ensembl annotation version 98; Cunningham *et al*, 2019). We further filtered out cells identified as outliers based on the median absolute deviation of > 3 from the median value of total UMI counts, total expressed genes and proportion of mitochondrial/nuclear genes expression using scater R package (version 1.18.2; McCarthy *et al*, 2017). Expression matrices were further normalized and integrated within sample group (LPS + PBS) using MNN algorithm implemented in scran R package (version 1.18.1; Lun *et al*, 2016). Secondary data analysis was done in BIOMEX software (Taverna *et al*, 2020). After batch correction, normalized data were auto scaled and principal component analysis was performed on all genes, followed by UMAP to construct a two‐dimensional representation of the data.

To unbiasedly group HSCs, we performed PCA, and used graph‐based clustering as implemented in the FindClusters function of the Seurat package (Satija *et al*, 2015). Cluster results were visualized using UMAP. Over‐partitioned clusters that represented the same biological phenotype were merged into a single cluster. We then used a two‐step approach to obtain ranked marker gene lists for each cluster. As a first criterion, marker genes for a given cluster should have the highest expression in that cluster compared to all other clusters and are therefore uniquely assigned to one cluster. Second, we ranked marker genes using a product‐based meta‐analysis (Hong *et al*, 2006). Briefly, we performed pair‐wise differential analysis of all clusters against all other clusters separately and ranked the results of each pair‐wise comparison by log_2_ fold change. The most upregulated genes received the lowest rank number (top ranking marker genes) and the most downregulated genes received the highest rank number.

Heatmap analysis was done using the heatmaply package (version 0.15.2), based on cluster‐averaged gene expression to account for cell‐to‐cell transcriptomic stochastics. Data were auto scaled for visualization.

We used the SCORPIUS package (version 1.0.2) to place cells onto pseudotime trajectories (Cannoodt *et al*, 2016). Dimensionality reduction was performed using the reduce_dimensionality function (we used 3 dimensions, all other parameters were default). Individual HSCs from each cluster were subsequently placed onto linear pseudotime using the infer_trajectory function of the SCORPIUS package using default settings.

### Assay for transposase‐accessible chromatin with sequencing (ATAC‐seq)

Eight C57BL/6 mice (10–12 weeks old) were injected with LPS as described above and sacrificed 4 h after the injection. Additional 8 C57BL/6 were injected with PBS as a control and sacrificed 4 h later. Blood and BM cells were isolated and processed and granulocytic response was assessed by cell counts and FACS analysis. HSCs (Lin^−^ c‐Kit^+^ Sca1^+^ CD48^−^ CD150^+^) were sorted as described and pooled into PBS and LPS samples. Together 57,000 (LPS) and 53,000 (PBS) were sorted. The ATAC sequencing libraries were prepared using ATAC seq kit (Active Motif Carlsbad, CA, USA). The libraries were analyzed using Agilent 2100 Bioanalyzer and sequenced on NextSeq 500 sequencer using NextSeq 500/550 high output kit (both Illumina, San Diego, CA, USA) according to the manufacturer's protocol with read length of 50 and 26 cycles for cDNA fragment in paired‐end configuration.

### ATAC‐seq analysis

Raw fastq files were analyzed using nf‐core/atacseq pipeline (Ewels *et al*, 2020; version 1.2.1) using GRCm38 genome (Ensembl annotation version 102; Yates *et al*, 2020). GUAVA pipeline (McCarthy *et al*, 2017) was used for the initial steps of the ATAC‐seq data processing. First, the adapters were trimmed using cutadapt, then aligned using bowtie2 (Langmead & Salzberg, 2012), and converted into sorted, and indexed bam file using samtools (Danecek *et al*, 2021). The additional filtering of the bam file (e.g., removing duplicates, and blacklist reads) was done using Picard (http://broadinstitute.github.io/picard/) and samtools. GUAVA then corrected the bam file by shifting the alignment. The peak calling was done using MACS2 (Zhang *et al*, 2008) with nomodel and nolambda parameters. The q‐values of the strongest peaks in the close (± 2.5kbp) neighborhood of the TSS were taken into consideration. Then, the enrichments between LPS and PBS conditions were calculated by dividing the *q*‐value of LPS by PBS and visualized using boxplot for the differentially expressed genes identified using scRNA‐seq (Appendix Table S1). Outliers with TSS enrichment > 25 were removed. The data were then tested using Welch's *t*‐test independent samples with Bonferroni correction.

### Colony culture

Murine colony culture assays were performed using Methocult GF M3434 (Stemcell Technologies, Vancouver, BC, Canada). 100 CD201^+^ or CD201^−^ HSCs were sorted and plated. Colonies were counted and cells harvested after 7 days of *in vitro* culture. Counting was performed by investigators blinded to the genotype. 5 × 10^4^ cells were stained for flow cytometry analysis; 3 × 10^3^ cells were spun on a slide and the remaining cells were lysed with 350 μl RLT buffer.

### Morphological analysis and differential counting

Morphological analysis and manual leukocyte differential counts of BM cells was performed using May‐Grünwald Giemsa stained cytospins. A minimum of 200 cells was analyzed.

### RNA isolation, cDNA preparation and quantitative RT–PCR

RNA from murine BM cells cultured in Methocult M3434, or sorted HSCs was extracted with RNeasy Micro Kit (Qiagen, Germantown, Maryland, USA) or Tri Reagent RT (Molecular Research Center, Cincinnati, OH, USA) and treated with DNaseI (Thermo Fisher Scientific, Waltham, MA, USA) according to manufacturer's instructions. Briefly, cDNA was prepared using SuperScript II Reverse Transcriptase (Thermo Fisher Scientific, Waltham, MA, USA). Quantitative RT–PCR was performed using a LightCycler® 480 SYBR Green I Master mix and samples were run on a LightCycler® 480 Instrument II (both Roche Molecular Systems, Pleasanton, CA, USA). For each sample, transcript levels of tested genes were normalized to *Actb*. Primer sequences used for quantitative RT–PCR are listed in below:

**Table:** 

Gene	Orientation	Organism	Sequence (5′ – 3′)
*Ela2*	F	Mouse	ACTCTGGCTGCCATGCTACT
	R	Mouse	GCCACCAACAATCTCTGA
*Mmp9*	F	Mouse	ACGGTTGGTACTGGAAGTTCC
	R	Mouse	CCAACTTATCCAGACTCCTGG
*Cebpe*	F	Mouse	AAGGCCAAGAGGCGCATT
	R	Mouse	CGCTCGTTTTCAGCCATGTA
*Mpo*	F	Mouse	GGAAGGAGACCTAGAGG
	R	Mouse	TAGCACAGGAAGGCCAAT
*Actb*	F	Mouse	GATCTGGCACCACACCTTCT
	R	Mouse	GGGGTGTTGAAGGTCTCAAA
*Cebpb*	F	Mouse	AAGCTGAGCGACGAGTACAAGA
	R	Mouse	GTCAGCTCCAGCACCTTGTG
*Il6*	F	Mouse	GACCTGTCTATACCACTTCA
	R	Mouse	GCATCATCGTTGTTCATA
*Bcl3*	F	Mouse	GTGGATGAGGATGGAGACA
	R	Mouse	AGGCTGAGTATTCGGTAGAC

### HSC differentiation in liquid culture

300 CD201^+^ or CD201^−^ HSCs were sorted and plated in 150 μl of StemSpan SFEM (Stemcell Technologies, Vancouver, BC, Canada) supplemented with 100 ng/ml SCF, 6 ng/ml IL3 and IL6 Peprotech (Rocky Hill, NJ, USA). Cells were counted and analyzed after 7 days of *in vitro* culture.

### BM transplantation assays

700 CD201^+^ or CD201^−^ HSCs were sorted from C57BL/6 (Ly5.2^+^) WT mice and mixed with 0.5 × 10^6^ whole BM cells isolated from C57BL/6 (Ly5.1^+^) mice and transplanted intravenously into lethally irradiated recipient C57BL/6 (Ly5.1^+^) mice. PB and BM of recipient mice were analyzed 16 weeks post‐transplantation. Cells were stained with Ly5.1 and Ly5.2 antibodies to distinguish donor and support cells as well as with B220, CD3, CD11b and Gr1 to determine tri‐lineage reconstitution, Lin, c‐Kit, Sca‐1, CD135, CD48, and CD150 were used to analyze MPP subsets, and Gr1, CD11b, and Ly6G to analyze granulocytic response. Recipients were 8–12 weeks old at the time of transplantation. A mouse was considered as responder when engraftment in BM LKS was > 0.5% of donor derived Lin^−^ cells 16 weeks after transplantation.

### Blood serum collection and G‐CSF level assessment by ELISA

Peripheral blood was collected from retro‐orbital vein of PBS or LPS injected mice and left for 30 min undisturbed at room temperature to coagulate. The clot was removed by centrifugation (5,000 rpm, 10 min, 4°C). The supernatant was transferred into a new tube and centrifuged again (12,000 rpm, 10 min, 4°C). The supernatant was transferred into a new tube one more time and the samples snap frozen by liquid nitrogen. G‐CSF levels were assessed by Mouse G‐CSF Quantikine ELISA Kit according to manufacturer's instruction.

### Statistical analysis

Statistical significance for indicated data sets was determined using two‐sided, unpaired Student's *t*‐test. Statistical analysis of ATAC‐seq data was done by using Welch's *t*‐test independent samples with Bonferroni correction. *P*‐values < 0.05 were considered statistically significant. GraphPad Prism outlier calculator was employed to exclude outliers based on Grubbs' test.

## Author contributions

**Karolina Vanickova:** Data curation; formal analysis; funding acquisition; investigation; visualization; methodology; writing – original draft; writing – review and editing. **Mirko Milosevic:** Formal analysis; methodology; writing – review and editing. **Irina Ribeiro Bas:** Investigation; writing – review and editing. **Monika Burocziova:** Investigation; writing – review and editing. **Asumi Yokota:** Formal analysis; funding acquisition; investigation; methodology; writing – review and editing. **Petr Danek:** Investigation; writing – review and editing. **Srdjan Grusanovic:** Investigation; writing – review and editing. **Mateusz Chiliński:** Formal analysis; methodology; writing – review and editing. **Dariusz Plewczynski:** Supervision; funding acquisition; writing – review and editing. **Jakub Rohlena:** Formal analysis; methodology; writing – review and editing. **Hideyo Hirai:** Formal analysis; supervision; funding acquisition; methodology; writing – review and editing. **Katerina Rohlenova:** Formal analysis; supervision; funding acquisition; methodology; writing – review and editing. **Meritxell Alberich‐Jorda:** Conceptualization; supervision; funding acquisition; methodology; writing – original draft; project administration; writing – review and editing.

## Disclosure and competing interests statement

The authors declare that they have no conflict of interest.

## Supporting information



Appendix S1

Source Data for Figure 1

Source Data for Figure 2

Source Data for Figure 3

Source Data for Figure 4

Source Data for Figure 5

Source Data for Figure 6

## Data Availability

scRNA‐seq data are available at Array Express under accession number E‐MTAB‐12324 (https://www.ebi.ac.uk/biostudies/arrayexpress/studies/E‐MTAB‐12324?key=a5fadbc0‐68bd‐4493‐b86c‐52e6ce0de39a), and ATAC‐seq data are available at Array Express under accession number E‐MTAB‐12310 (https://www.ebi.ac.uk/biostudies/arrayexpress/studies/E‐MTAB‐12310?key=10dc5da4‐b1a9‐4aa1‐acdb‐1ede8bfbfaab).
