# Supplementary material for: Hematopoietic stem cells undergo a lymphoid to myeloid switch in early stages of emergency granulopoiesis
Source: EMBO J. 2023 Oct 17;42(23):e113527. doi: 10.15252/embj.2023113527 (PMC10690458; doi:10.15252/embj.2023113527)
Supplement: Supplementary file 1 — Appendix S1 [file EMBJ-42-e113527-s006.pdf]

Contents

Appendix Figure S1 ………………………………………………………………….. 2

Appendix Figure S2 ………………………………………………………………….. 3

Appendix Figure S3 ………………………………………………………………….. 4

Appendix Figure S4 ………………………………………………………………….. 5

Appendix Table S1 …………………………………………………………………... 6

Appendix Table S2 …………………………………………………………………... 7

Appendix Figure S1

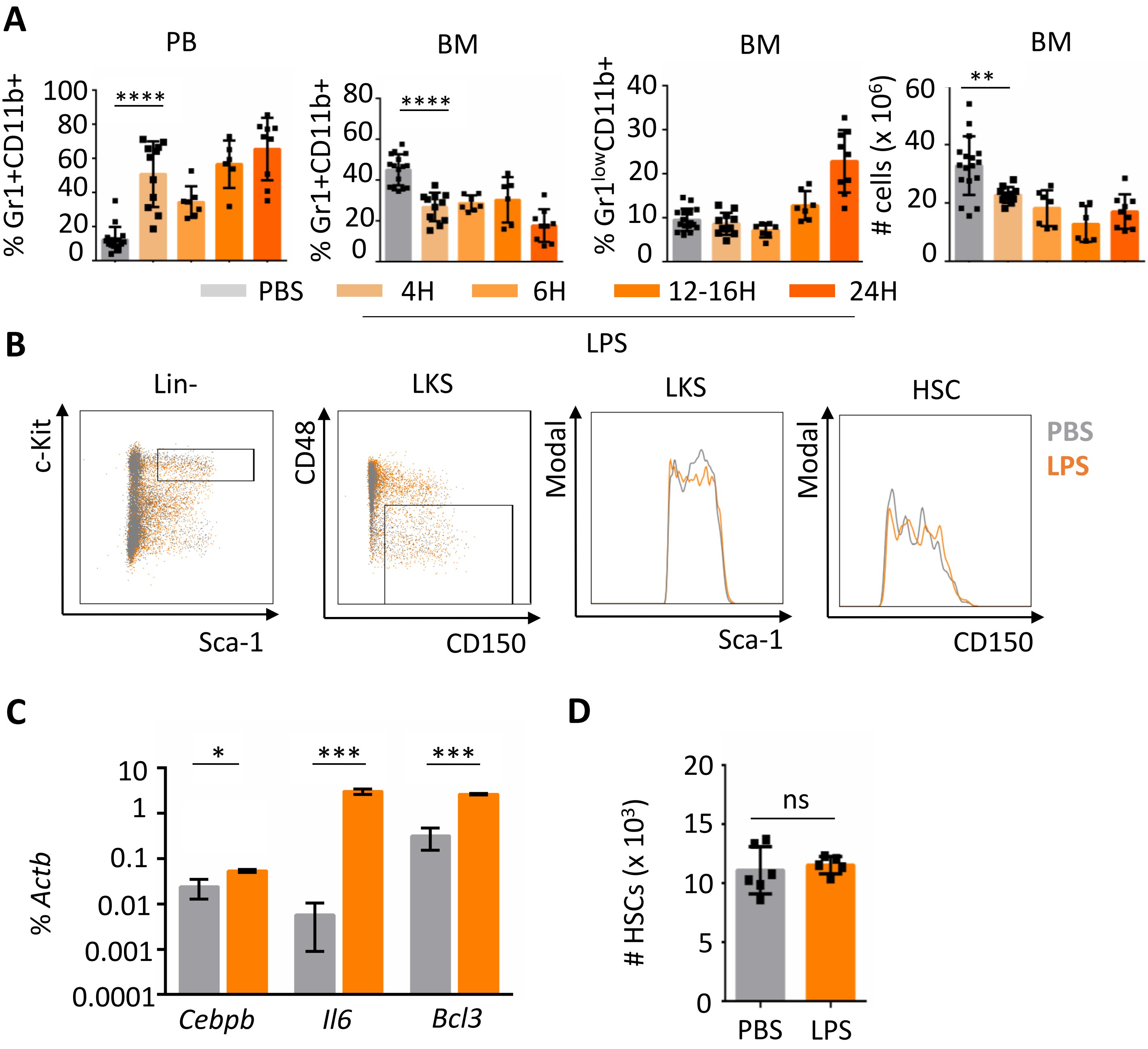

Appendix Figure S1. Emergency granulopoiesis response upon LPS stimulation. (A) Peripheral blood (PB) and bone marrow (BM) analysis 4, 6, 12-16 or 24 hours after 35 µg LPS (shades of orange) or PBS control (gray) injection into C57Bl/6 mice. Each symbol represents values for one mouse. Data represents mean ± SD from 4 independent experiments. **(B)** Flow cytometric analysis of BM cells isolated from PBS- (gray) or LPS- (orange) treated mice. Left dot plots illustrate expression of c-Kit and Sca-1 in the Lin- BM cells, and CD48 and CD150 expression in the LKS population. Right histograms indicate expression of Sca-1 in the LKS population and CD150 expression in the HSC population. **(C)** Quantitative RT-PCR from HSCs isolated from C57Bl/6 mice treated with PBS (gray) or 35 µg LPS (orange) for 4 hours. Expression of *Cebpb*, *Il6*, and *Bcl3* is indicated. The y-axes represent relative expression compared with *Actb* control. Data represent mean ± SD from a representative experiment out of 2 independent experiments. **(D)** Number of HSCs in BM (leg and hip) 4 hours upon PBS or LPS administration. Y-axis indicates absolute number. Data represents mean ± SD from 2 independent experiments. In this figure, two-tailed Student's t test was used to assess statistical significance (*P < 0.05, **P < 0.01, ***P < 0.001, ****P < 0.0001, ns: not significant).

Appendix Figure S2

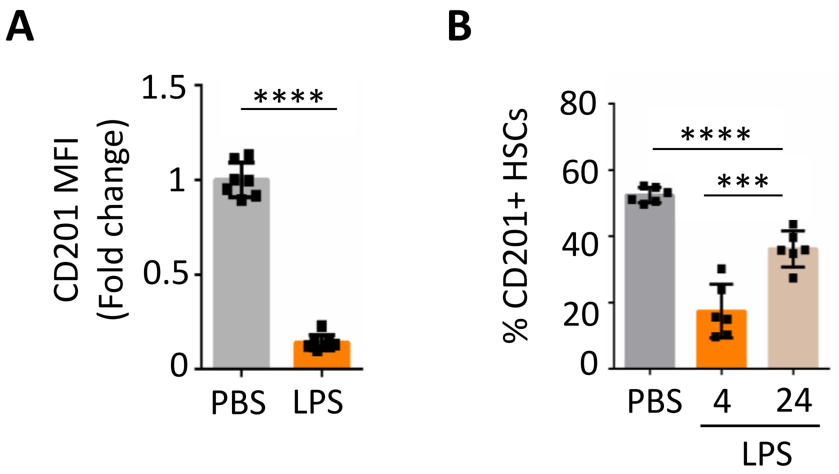

Appendix Figure S2. CD201 levels in HSCs upon LPS-induced emergency granulopoiesis. (A) CD201 in HSCs 4 hours after administration of LPS or PBS control. Values indicate CD201 mean fluorescence intensity (MFI) relative to PBS treatment. X-axis indicates fold change. **(B)** Percentage of CD201+ HSCs in BM upon administration of PBS or LPS. X-axis indicates time of analysis upon LPS administration. Data represents mean ± SD from 2 independent experiments. Each symbol represents values for 1 mouse. At least 6 mice were used in each group. Two-tailed Student's t test was used to assess statistical significance (***P < 0.001, ****P < 0.0001).

Appendix Figure S3

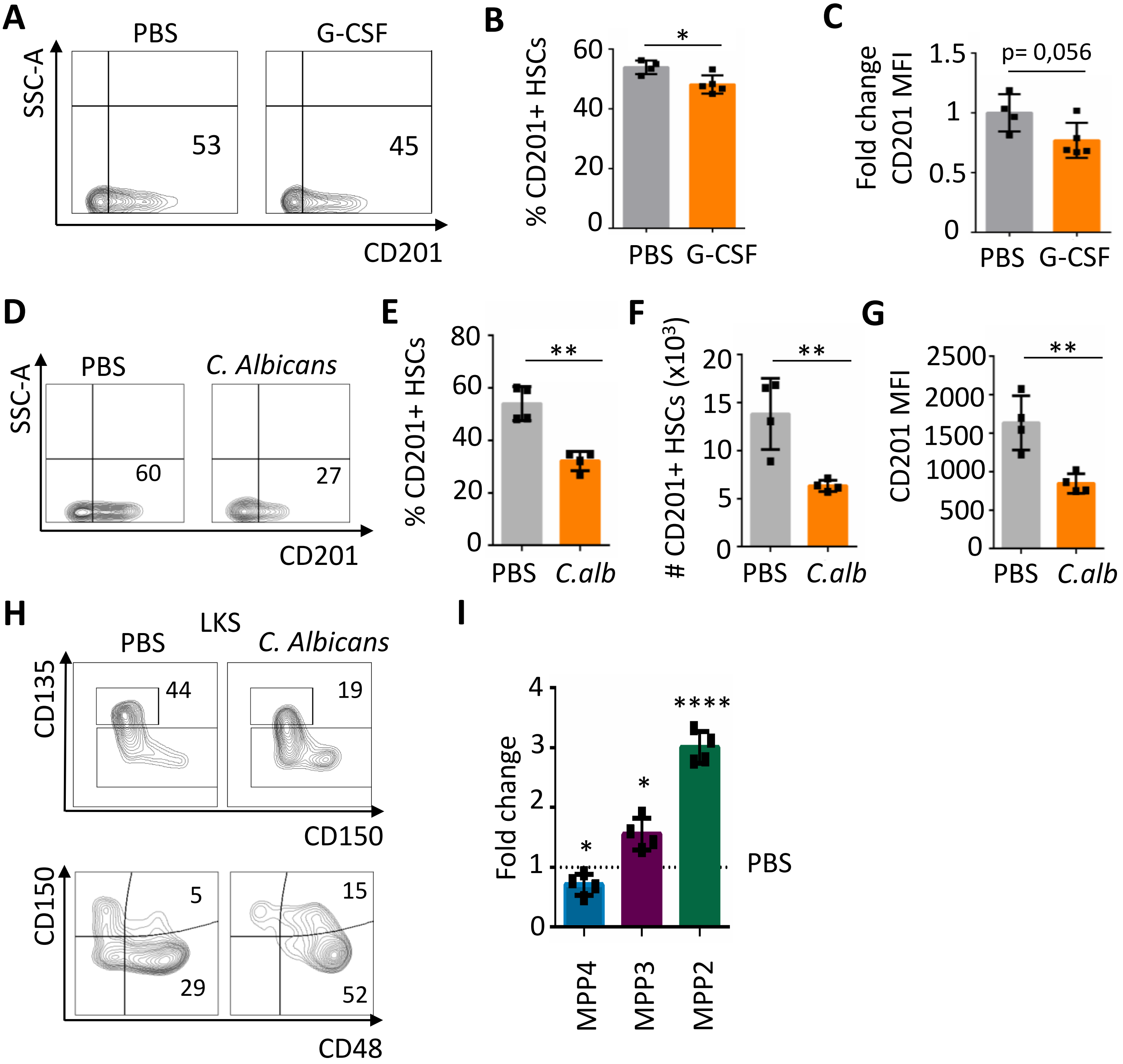

Appendix Figure S3. CD201 levels in HSCs during emergency granulopoiesis induced by administration of G-CSF and *Candida albicans (C. albicans)*. (A) Representative flow cytometric plots of BM HSCs isolated from mice treated with PBS control or 250 µg/kg G-CSF (intraperitoneally) for 4 hours. X-axes indicate expression of CD201 in HSCs. Numbers indicate percentage of CD201+ HSCs. **(B-C)** Quantification of panel a. Panel b indicates percentage of CD201+ HSCs upon treatment, and panel c indicates the mean fluorescence intensity (MFI) relative to PBS treatment. **(D)** Representative flow cytometric plots of HSCs isolated from PBS or *C. albicans* treated mice. X-axes indicate CD201 expression in HSCs. Numbers indicate percentage of CD201+ HSCs.**(E-G)** Quantification of panel d. Panel e indicates percentage of CD201+ HSCs, panel f number of CD201+ HSCs, and panel g indicates the MFI relative to PBS treatment. **(H)** Representative plots from BM isolated from mice challenged with PBS (left) or *C. albicans* (right). Plots indicate gating strategy to identify progenitor and stem cell populations. Numbers indicate percentages of LKS. **(I)** Quantification of panel h. Y-axes indicate fold change from PBS- to *C. albicans*-treated mice. In this figure, at least 4 animals were included in each group. All data represent mean ± SD from 2 independent experiments. Two-tailed Student's t test was used to assess statistical significance (*P < 0.05, **P < 0.01, and ****P < 0.0001).

Appendix Figure S4

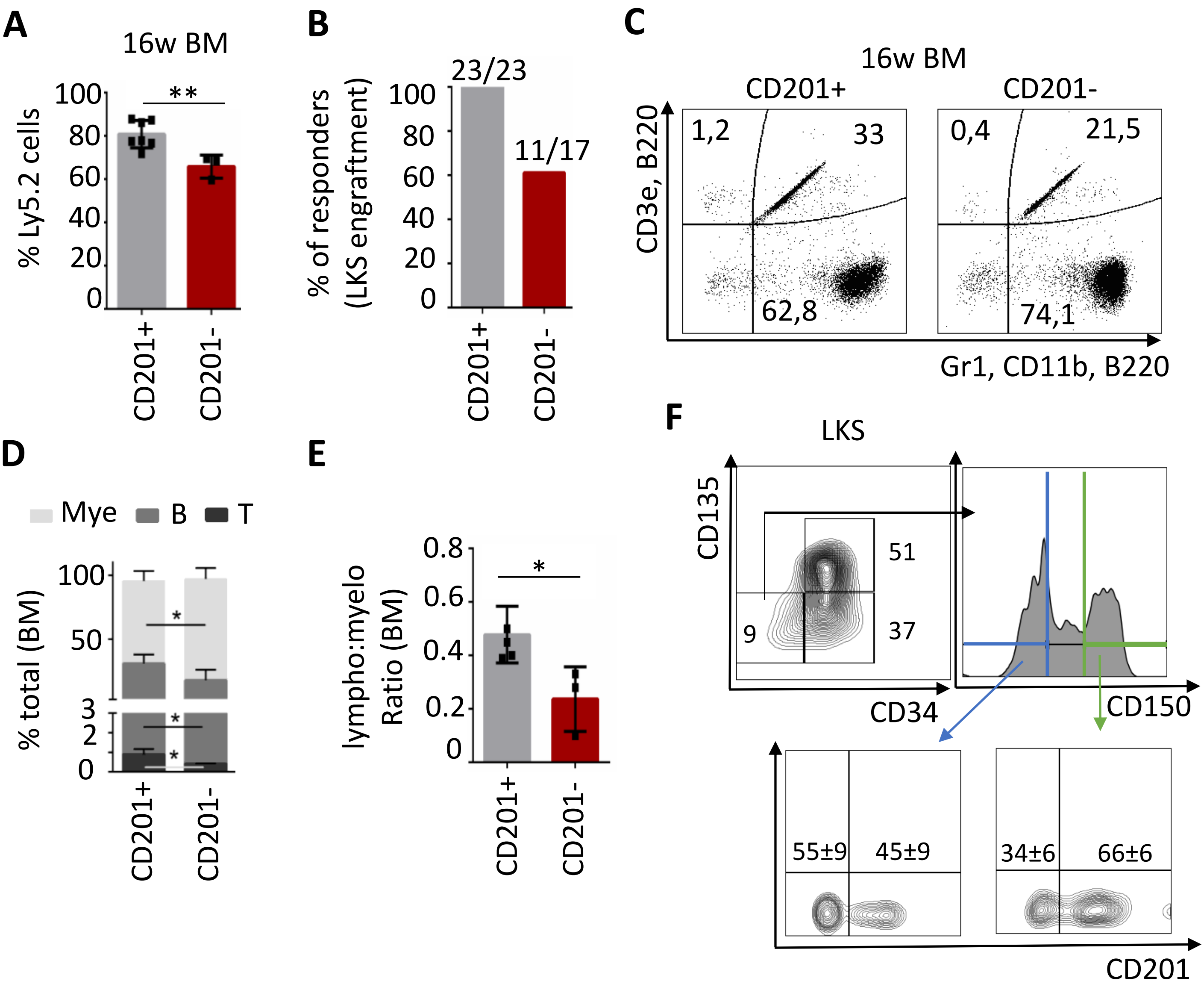

Appendix Figure S4. CD201+ and CD201- HSC output in steady-state conditions. (A) BM analysis of mice transplanted with CD201+ or CD201- HSCs. Y-axis indicates percentage of Ly5.2+ donor derived CD201+ or CD201- HSCs in BM 16 weeks after transplantation. X-axis indicates group transplanted with CD201+ or CD201- HSCs. This is a representative experiment out of 3. Each symbol indicates one mouse. **(B)** Quantification of the number of animals that exhibited engraftment upon transplantation. A mouse was considered as a responder when engraftment in BM LKS was > 0. 5 % of donor derived Lin- cells 16 weeks after transplantation. Y-axis indicates the percentage of responders in mice transplanted with CD201+ or CD201- HSCs. **(C)** Representative flow cytometric plots showing tri-lineage reconstitution in BM 16 weeks upon transplantation of CD201+ and CD201- HSCs. The x-axes indicate expression of Gr1, CD11b, and B220. The y-axes indicate expression of CD3e and B220. Upper left quadrant indicates percentage of T cells, upper right quadrant indicates percentage of B cells, and lower right quadrant indicates percentage of myeloid cells. **(D)** Quantification of panel c. At least 5 animals were included in each group. **(E)** Quantification of lymphoid versus myeloid derived cells upon transplantation of CD201+ and CD201- HSCs. Y-axis indicates the lymphoid to myeloid ratio in BM 16 weeks upon transplantation. Data in this figure represent mean ± SD from 2 independent experiments. Two-tailed Student's t test was used to assess statistical significance (*P < 0.05, *P < 0.01). **(F)** Representative flow cytometry plots based on expression of CD135 and CD34 in LKS cells (left panel). CD135-CD34- cells were gated into CD150- (blue gate) and CD150 (green gate), and further divided according to CD201 expression levels (lower plots). Numbers indicate average ± standard deviation (n=5 mice).

Appendix Table S1

Rank	Meg/E	Transition	Lymphoid	Inflamm	Myeloid
1	Hmgb2	Cmtm7	Hlf	Iigp1	Ccl5
2	Top2a	Gpx1	Prtn3	Cxcl10	Rsad2
3	Stmn1	Coro1a	Lmo2	Ly6a	Isg15
4	Dut	Prdx2	Txnip	Gbp2	Nfkbia
5	Mki67	Park7	Gcnt2	Gm4951	Fyb
6	Ran	Igfbp4	Adgrg1	Ifit3	Marcks
7	Pf4	Gpi1	Mllt3	Serpina3g	Neurl3
8	Cks2	Fkbp3	Cbfa2t3	Stat1	Gbp3
9	Tgfb1	Aprt	Hacd4	Plac8	Cd74
10	Tuba1b	Aldh2	Lst1	Rgs1	Gadd45b
11	Tubb5	Lyz2	Adgrl4	Slfn5	Ifit1
12	Hmgb1	Taldo1	Angpt1	Ifi203	Tnip3
13	Lmnb1	Pgam1	Mettl7a1	Igtp	Batf
14	Dctpp1	Myc	Eif4a2	Mndal	Manf
15	Nap1l1	Vim	Procr	Ifi44	Hspa5
16	Pbx1	Nme4	Arhgdib	Ifi204	Ccl4
17	Ldha	Tecr	Sox4	Socs1	Fth1
18	Anp32b	Trmt112	Smarca2	Gbp7	Oasl1
19	Nrgn	Pebp1	Zfp36l2	Zbp1	Traf1
20	Nucks1	Lamtor4	Zbtb20	Gbp5	Cebpd
21	Cdk6	Emp3	Ptpn18	Gbp4	Sod2
22	Cdca8	Vamp8	Msi2	Serpina3f	Ikbke
23	Tmpo	Pkm	Nfic	Ms4a4c	Spi1
24	Cks1b	Tspan4	Ramp1	Cxcl9	Cd69
25	Ptma	Gsto1	Pdzk1ip1	Irf7	Icosl
26	Atpif1	Ptov1	Pik3ip1	Isg20	Cxcl2
27	Pcna	Gclm	Ypel3	Bst2	Pdia6
28	Smc2	Phgdh	Gimap1	Pim1	Nfkb1
29	Apoe	H2-DMa	Pik3r1	Samhd1	Mapkapk2
30	Birc5	Ech1	Cox7a2l	Jun	Cebpb
31	Cdk1	Rangrf	Kit	Ifi47	Pou2f2
32	Smc4	Pfkl	Sirt3	Ifit2	Tm4sf1
33	Ybx3	Slc25a4	Foxp1	Herc6	Cmpk2
34	Spc24	Mettl26	Smpdl3a	Irf1	Btg1
35	Lig1	Adh5	Ptprcap	Cd53	Sdc4
36	Snrpd1	Imp3	Glul	Ifitm1	Ifih1
37	Dnmt1	Rps26	Ogt	Shisa5	Ncf1
38	Itga2b	Fkbp1a	Zfp608	Oasl2	Herpud1
39	Rrm1	Gmfg	Akap13	Tnfsf10	Milr1
40	Mcm3	Ctsd	Tbxas1	Pnp	Lsp1
41	Tyms	Ccdc107	Nceh1	Junb	Ptms
42	Pclaf	Dtd1	Ptpre	Trim30a	Clic4
43	Atad2	Scp2	Mecom	Apobec3	Lcn2
44	Slc25a5	Rpl32	Sptssa	Ifitm3	Parp14
45	Hdgf	Naca	Ldhb	Epsti1	Slfn2
46	Dtymk	Mrpl52	Sptbn1	Selp	Hspa8
47	Nasp	Tmem205	Tsc22d3	Socs3	Eif1
48	Dek	Matk	Klhl24	Ppa1	Ebi3
49	Gmnn	Il11ra1	Alox5ap	Irgm1	Rasip1
50	Hmgb3	Abhd14a	Atp2b4	Ly6e	Serp1

Appendix Table S1. List of Top50 genes differentially expressed in the 5 distinct sub-clusters.

Appendix Table S2

Myeloid	Lymphoid
Hdc	Flt3
Csf3r	Tcf3
Irf8	Notch1
Nr4a1	Zbtb1
Ccl5	Satb1
Cebpb	Ikzf1
Cebpd	Egr1

Appendix Table S2. List of genes used to define the myeloid-biased or lymphoid-biased HSCs in Figure 1g.
