# Supplementary figures and images for: Hematopoietic stem cells undergo a lymphoid to myeloid switch in early stages of emergency granulopoiesis
Source: EMBO J. 2023 Oct 17;42(23):e113527. doi: 10.15252/embj.2023113527 (PMC10690458; doi:10.15252/embj.2023113527)

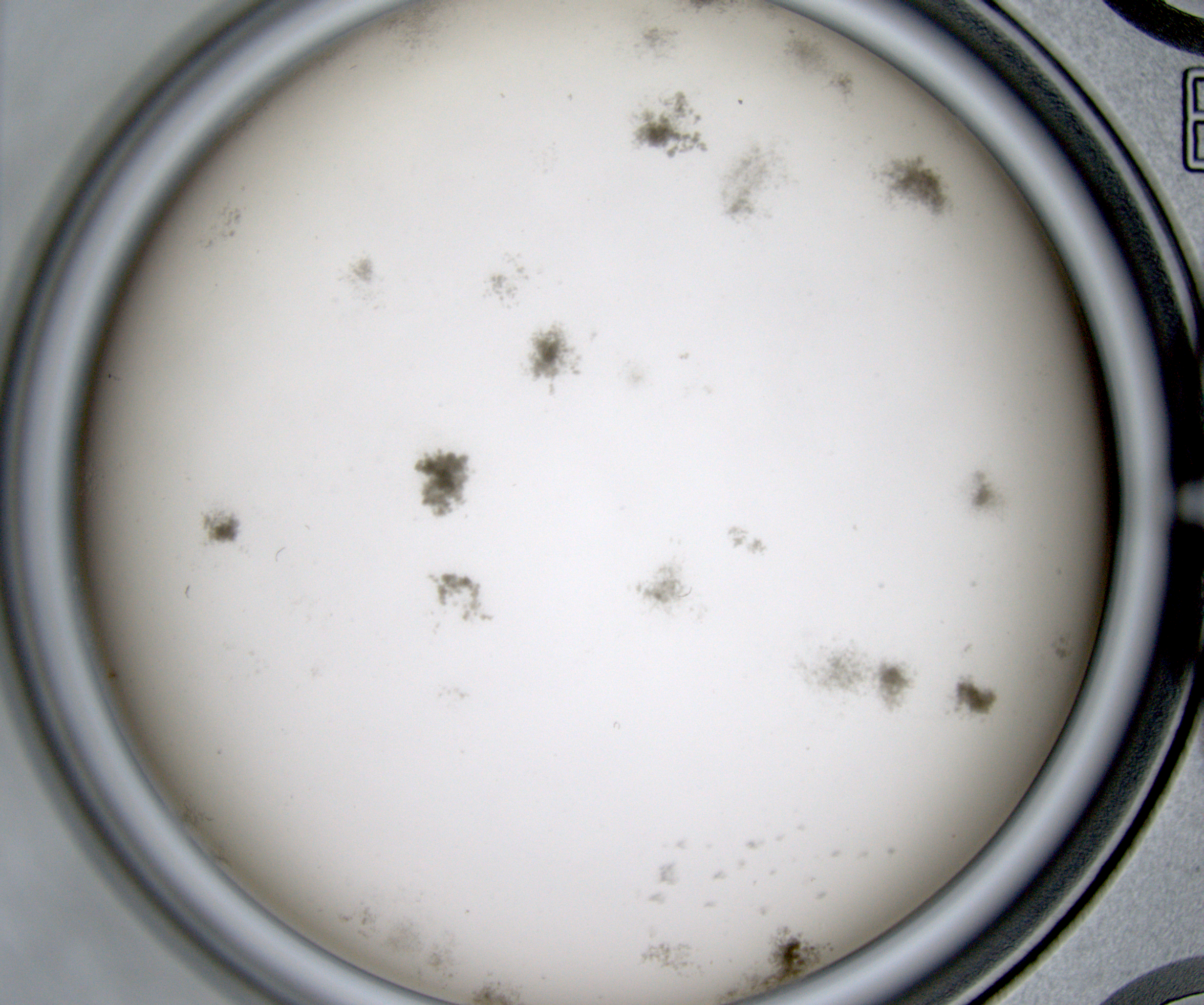

Supplement: Supplementary file 4 — Source Data for Figure 3 [file EMBJ-42-e113527-s003.zip › Figure_3/3A/CD201+a.tif]

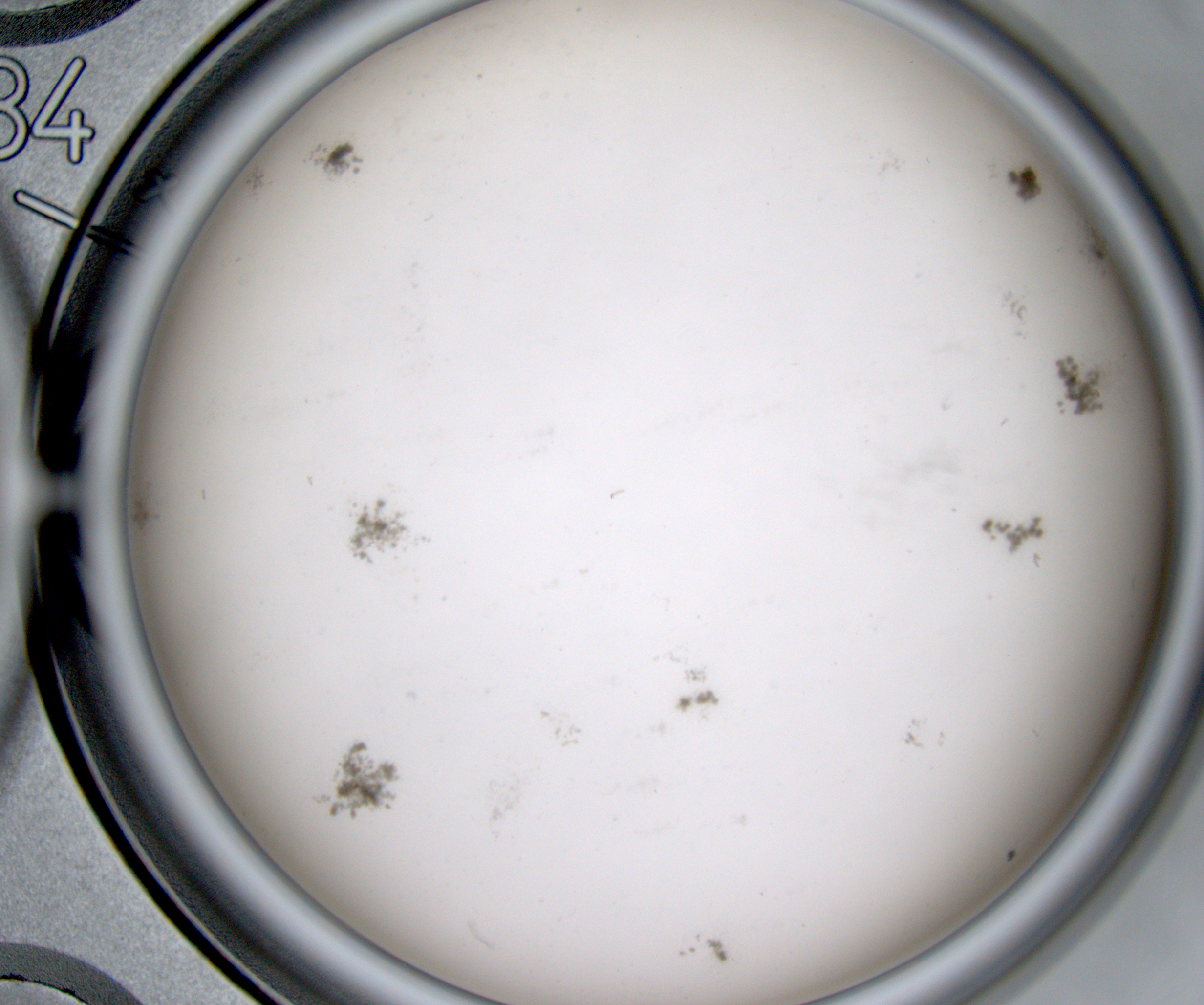

Supplement: Supplementary file 4 — Source Data for Figure 3 [file EMBJ-42-e113527-s003.zip › Figure_3/3A/CD201-b.tif]

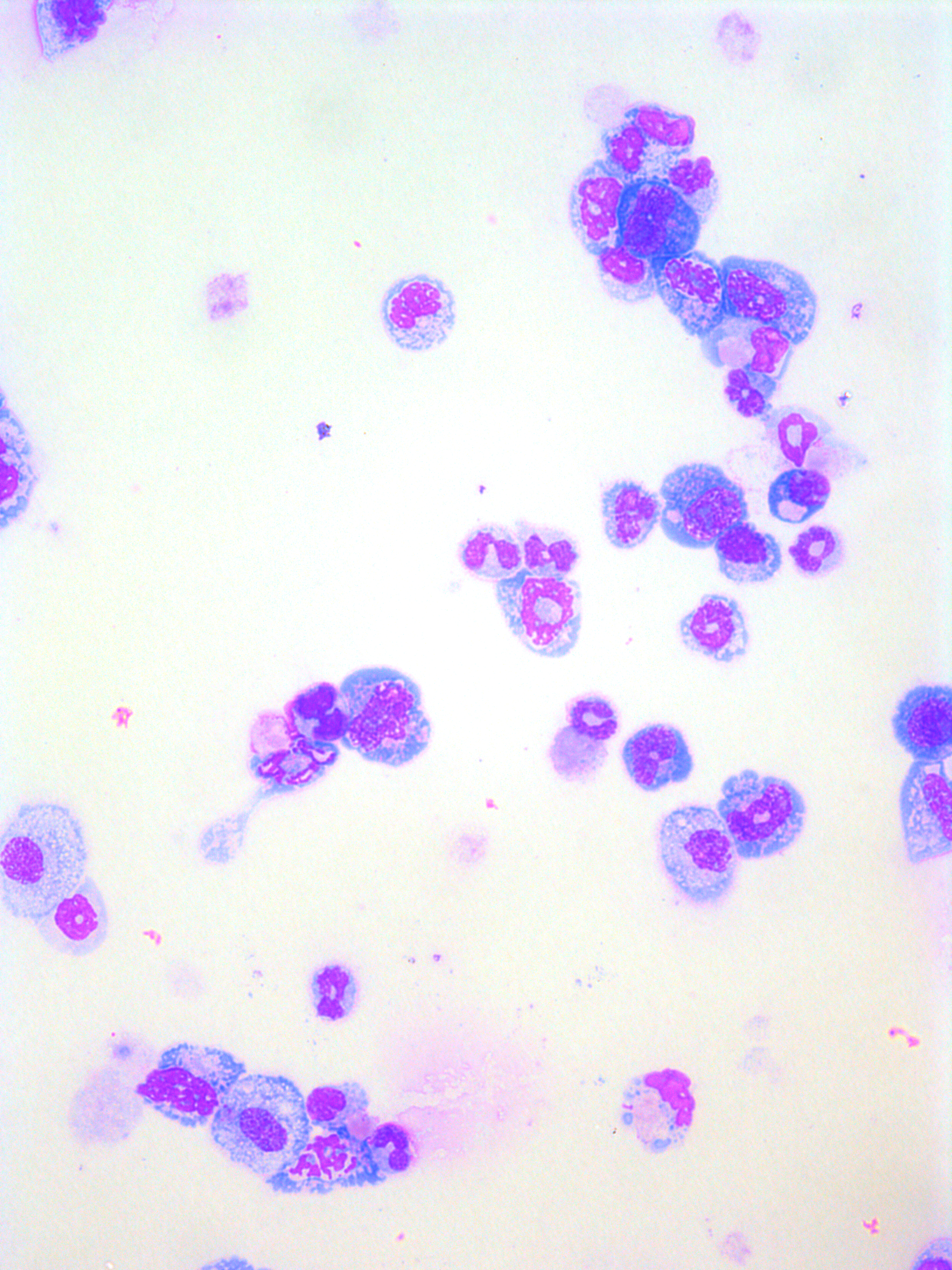

Supplement: Supplementary file 4 — Source Data for Figure 3 [file EMBJ-42-e113527-s003.zip › Figure_3/3D/CD201+_b_.tif]

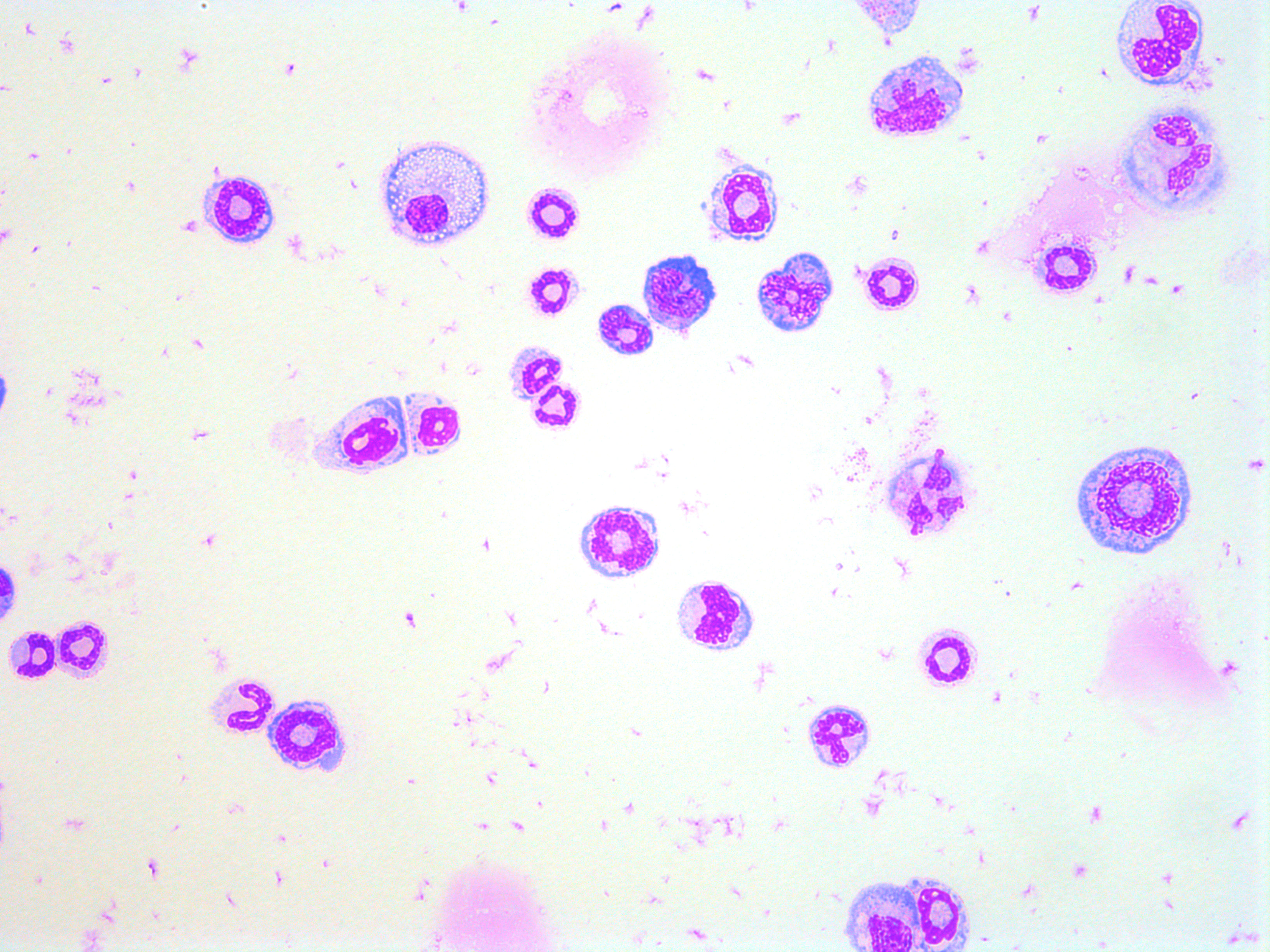

Supplement: Supplementary file 4 — Source Data for Figure 3 [file EMBJ-42-e113527-s003.zip › Figure_3/3D/CD201-_a_.tif]
