# Supplementary material for: Hematopoietic stem cells undergo a lymphoid to myeloid switch in early stages of emergency granulopoiesis
Source: EMBO J. 2023 Oct 17;42(23):e113527. doi: 10.15252/embj.2023113527 (PMC10690458; doi:10.15252/embj.2023113527)
Supplement: Supplementary file 8 [file EMBJ-42-e113527-s004.pdf]

THE EMBO JOURNAL

Hematopoietic stem cells undergo a lymphoid to myeloid switch in early stages of emergency granulopoiesis

Karolina Vanickova, Mirko Milosevic, Irina Ribeiro Bas, Monika Burocziova, Asumi Yokota, Petr Danek, Srdjan Grusanovic, Mateusz Chilinski, Dariusz Plewczynski, Jakub Rohlena, Hideyo Hirai, Katerina Rohlenova, and Meritxell Alberich Jorda
DOI: 10.15252/embj.2023113527

Corresponding author(s): Meritxell Alberich Jorda (alberich@img.cas.cz)

Review Timeline:

Submission Date:	16th Jan 23
Editorial Decision:	9th Mar 23
Revision Received:	15th Aug 23
Editorial Decision:	15th Sep 23
Revision Received:	20th Sep 23
Accepted:	25th Sep 23

Editor: Karin Dumstrei

Transaction Report:

(Note: With the exception of the correction of typographical or spelling errors that could be a source of ambiguity, letters and reports are not edited. Depending on transfer agreements, referee reports obtained elsewhere may or may not be included in this compilation. Referee reports are anonymous unless the Referee chooses to sign their reports.)

Dear Meritxell,

Thank you for submitting your manuscript to The EMBO Journal. Your study has now been seen by two referees and their comments are provided below.

As you can see from the comments, the referees find that analysis interesting, but also consider that further work is needed to consider publication here. In particular we would needed insight into the molecular mechanism promoting CD201 downregulation upon LPS exposure. We would also need some further in vivo work to support the key findings.

Should you be able to address the raised issues then I would like to invite you to submit a revised version. It would be good to discuss the revision points further and I am available to do so via email or video.

When preparing your letter of response to the referees' comments, please bear in mind that this will form part of the Review Process File, and will therefore be available online to the community. For more details on our Transparent Editorial Process, please visit our website: https://www.embopress.org/page/journal/14602075/authorguide#transparentprocess

I thank you for the opportunity to consider your work for publication. I look forward to discussing the revisions further with you

Yours sincerely,

Karin

Karin Dumstrei, PhD
Senior Editor
The EMBO Journal

Instructions for preparing your revised manuscript:

Please make sure you upload a letter of response to the referees' comments together with the revised manuscript.

Please also check that the title and abstract of the manuscript are brief, yet explicit, even to non-specialists.

When assembling figures, please refer to our figure preparation guideline in order to ensure proper formatting and readability in print as well as on screen:
https://bit.ly/EMBOPressFigurePreparationGuideline
See also guidelines for figure legends: https://www.embopress.org/page/journal/14602075/authorguide#figureformat

At EMBO Press we ask authors to provide source data for the main manuscript figures. Our source data coordinator will contact you to discuss which figure panels we would need source data for and will also provide you with helpful tips on how to upload and organize the files.

IMPORTANT: When you send the revision we will require
- a point-by-point response to the referees' comments, with a detailed description of the changes made (as a word file).
- a word file of the manuscript text.
- individual production quality figure files (one file per figure)
- a complete author checklist, which you can download from our author guidelines (https://www.embopress.org/page/journal/14602075/authorguide).
- Expanded View files (replacing Supplementary Information)
Please see out instructions to authors
https://www.embopress.org/page/journal/14602075/authorguide#expandedview

Please remember: Digital image enhancement is acceptable practice, as long as it accurately represents the original data and conforms to community standards. If a figure has been subjected to significant electronic manipulation, this must be noted in the figure legend or in the 'Materials and Methods' section. The editors reserve the right to request original versions of figures and the original images that were used to assemble the figure.

Further information is available in our Guide For Authors: https://www.embopress.org/page/journal/14602075/authorguide

We realize that it is difficult to revise to a specific deadline. In the interest of protecting the conceptual advance provided by the

work, we recommend a revision within 3 months (7th Jun 2023). Please discuss the revision progress ahead of this time with the editor if you require more time to complete the revisions.

As a matter of policy, competing manuscripts published during this period will not negatively impact on our assessment of the conceptual advance presented by your study. However, we request that you contact the editor as soon as possible upon publication of any related work, to discuss how to proceed.

Use the link below to submit your revision:

https://emboj.msubmit.net/cgi-bin/main.plex

Referee #1:

General comment
In their study, Vanickova et al addressed the contribution of hematopoietic stem cells (HSCs: Lin- Sca1+ cKit+ CD48- CD150+) to emergency granulopoiesis by exposing mice to LPS and assessing the single-cell transcriptomic alterations in sorted HSCs 4 hours later. In agreement with previous studies (PMID: 29198940 ; PMID: 32169166), the authors show that LPS exposure induced a myeloid different bias in HSCs while reducing the lymphoid-primed HSCs. Using single-cell RNA data the authors identified CD201, a previously known HSC surface marker (PMID: 16304059 ; PMID: 25892186) to be enriched in lymphoid-biased HSCs in steady-state conditions. Interestingly the authors show that CD201+ HSCs are still active contributors to granulopoiesis upon LPS exposure, thus establishing a cellular basis for the observed increased HSC myeloid bias at the expense of lymphoid output.

Overall, this is an interesting and well-preformed study that establishes the cellular dynamics underlying LPS-induced lymphoid to myeloid differentiation bias and identifies a novel marker that enriches for lymphoid-biased HSCs. However, the novelty of the study is limited, as multiple aspects of the study have been previously established (see above). Moreover there are specific experimental aspects that should be improved (see bellow).

Major concerns:
1. It is unclear if LPS-mediated myelopoiesis in CD201+ HSCs is acting directly or indirectly via inflammatory signaling. This could be clarified by transplanting TLR4 KO CD201+ and CD201- HSCs and expose them to LPS, further checking myelopoietic response. Additionally, It would be important to determine the TLR4 expression levels on CD201+ and - HSCs. Resolving this aspects would be important in order to address the molecular pathway mediating CD201 downregulation downstream of LPS/inflammatory signaling.

2. While it is clear that CD201+ HSC generate CD201- cells upon transplantation and LPS treatment, whether CD201- HSCs can regenerate the CD201+ HSC pool at later time points post LPS treatment should be determined. For this purpose transplantation of CD201- HSCs followed by LPS treatment should be preformed.

3. Ly-HSC and My-HSC have been previously defined based on CD150 expression levels (PMID: 20304793). How this relates with CD201 expression should be determined.

4. To increase the significance of the study, the contribution of CD201+ HSC to myelopoiesis should be performed in additional settings, particularly in a in vivo infection model. The data concerning G-CSF-mediated downregulation of CD201 expression in HSCs is insuficient (S2C and S2D).

Minor concerns:
1. The absolute number of HSCs after LPS exposure should be shown.
2. On page 7 I believe figure calling should read "2H-2J" instead of "3H-3J".

Referee #2:

Well-written and concise manuscript that provides relevant and novel observations on HSC biology: 1) CD201 (Procr) marks a population enriched in lymphoid-biased HSCs 2) Emergency granulopoiesis is characterized by a myeloid transcriptional switch in HSC and loss of CD201.

These observations have important implications as 1) Procr is currently considered a true HSC marker and 2) The myeloid switch that happens during emergency granulopoiesis within the HSC compartment encompasses the lymphoid-biased HSC compartment.

The experimental design is adequate and the methods state-of-the-art. The authors provide solid results with data that match the conclusions. The major limitation of this study is the lack of mechanistic insights.

There are a couple of major questions that would be critical to address to realize the full potential of the work, and other more minor aspects.

Specific major points:

1. The authors do not comment on it but it is surprising to see the virtually absent myeloid-biased HSC under PBS conditions. How do their annotated clusters and gene signatures compare to previously published observations?
2. How is LPS or G-CSF connected to the reduction in CD201? I understand the hurdles and limitations of performing functional studies targeting HSCs specifically, but some mechanistic insights would be required. Does Cebpb reduce the expression of CD201 in HSCs?
3. Is the loss of CD201 consequence or can it also contribute to the lymphoid to myeloid transcriptional switch? To rule out this question, the authors may want to perform a short term in vivo treatment with a blocking antibody.
4. Which subset CD201+ HSC or CD201- HSC performs better under emergency granulopoiesis or are they just the same? Are the dynamics of the process similar for both? The authors show a different response for MPP3 but more information would be relevant. Are they both really needed to respond under emergency granulopoiesis? One may think that a more adaptive response would involve increased granulocyte output without compromising the lymphoid output.
5. Do lymphoid-biased HSC regain CD201 expression under resting conditions following LPS challenge?

Specific minor points:

1. Figures 2H-2J are referenced in the text as 3H-3J. Please correct.
2. Under my opinion, study of the preferential lineage output of CD201+ HSC in vivo "under steady state conditions" would require lineage tracing. I suggest to rephrase the experiments using lethal irradiation and transplantation to "resting conditions following hematopoietic challenge" or similar.
3. Please provide a brief explanation of the pseudo-time analysis in the Results section.
4. Please reference Table S1 for better understanding of Figure 1H.
5. Indicate if the mice are SOPF, especially relevant in studies involving inflammation and myelopoiesis.
6. Are CD201- HSC able to generate CD201+ HSC?
7. Fig 1I, please show UMAP of both PBS and LPS separately as well. From this panel, it seems like CD201+ cells comprise a small subset within the lymphoid-biased HSC cluster. Please discuss.
8. It would be preferable to use FACS gating strategies with lymphoid and myeloid cell subsets separatetely.

Point-by-point letter to reviewers

Point-by-point response to reviewers' comments:

We appreciate the careful analysis of our manuscript by the reviewers and we want to thank them for the time they spent reviewing our manuscript. We believe that their constructive suggestions helped us to improve our manuscript. As requested by the reviewers, we have performed additional experiments and addressed their suggestions as described in detail below, mostly to (1) provide molecular insights into the downregulation and participation of CD201 in emergency granulopoiesis, and (2) elucidate the mechanisms that employ CD201+ and CD201- HSCs to contribute to emergency granulopoiesis. With these revisions, we believe that our manuscript has been substantially strengthened.

Reviewer Comments:

Referee #1:

General comment

In their study, Vanickova et al addressed the contribution of hematopoietic stem cells (HSCs: Lin- Sca1+ cKit+ CD48- CD150+) to emergency granulopoiesis by exposing mice to LPS and assessing the single-cell transcriptomic alterations in sorted HSCs 4 hours later. In agreement with previous studies (PMID: 29198940 ; PMID: 32169166), the authors show that LPS exposure induced a myeloid different bias in HSCs while reducing the lymphoid-primed HSCs. Using single-cell RNA data the authors identified CD201, a previously known HSC surface marker (PMID: 16304059 ; PMID: 25892186) to be enriched in lymphoid-biased HSCs in steady-state conditions. Interestingly the authors show that CD201+ HSCs are still active contributors to granulopoiesis upon LPS exposure, thus establishing a cellular basis for the observed increased HSC myeloid bias at the expense of lymphoid output.

Overall, this is an interesting and well-preformed study that establishes the cellular dynamics underlying LPS-induced lymphoid to myeloid differentiation bias and identifies a novel marker that enriches for lymphoid-biased HSCs. However, the novelty of the study is limited, as multiple aspects of the study have been previously established (see above). Moreover there are specific experimental aspects that should be improved (see bellow).

Major concerns:

1. It is unclear if LPS-mediated myelopoiesis in CD201+ HSCs is acting directly or indirectly via inflammatory signaling. This could be clarified by transplanting TLR4 KO CD201+ and CD201- HSCs and expose them to LPS, further checking myelopoietic response. Additionally, It would be important to determine the TLR4 expression levels on CD201+ and - HSCs. Resolving this aspects would be important in order to address the molecular pathway mediating CD201 downregulation downstream of LPS/inflammatory signaling.

We thank the reviewer for this remark which together with the following comments and the remarks from reviewer 2 helped us to gain important knowledge on the mechanistic insights which drive emergency granulopoiesis at the HSC level.

We hypothesized that emergency granulopoiesis might be activated in HSCs in a direct (LPS/TLR4) or indirect (G-CSF/G-CSF-R) manner. Of note, we observed that G-CSF levels are greatly increased 4 hours upon LPS injection, indicating that both activating mechanisms are potentially involved in the process. Thus, we investigated the mechanism driving emergency granulopoiesis on CD201+ and CD201- HSCs.

Interestingly, we observed that both CD201+ and CD201- HSCs rely on different signaling pathways under acute infection. While CD201+ HSCs express higher levels of TLR4 and have higher activation of NF-κB signaling upon *in vitro* LPS stimulation, the CD201- HSCs express higher levels of G-CSF-R and have higher activation of pSTAT3 signaling upon G-CSF-stimulation. Moreover, while the LIP isoform of C/EBPβ (important for cell proliferation) is present in both CD201+ and CD201- HSCs, the LAP/LAP* C/EBPβ isoforms (important for myeloid differentiation) are present only in the CD201- HSCs fraction (see new Figure 6).

If we incorporate these results to our previous observations, we conclude that emergency granulopoiesis is supported by a subpopulation of HSCs which upon pathogen sensing undergo a radical transcriptional rewiring that promotes their myeloid output. Initially, the pathogen is directly sensed by TLR4 on the surface of a steady state lymphoid-biased CD201+ HSCs, causing a rapid activation of the downstream NF-κB signaling pathway. Subsequently, the lymphoid-myeloid transcriptional switch, marked by the loss of CD201 expression, occurs and emergency granulopoiesis is then supported by myeloid-biased CD201- HSCs. CD201- HSCs respond to the infection in an indirect manner through G-CSF-R on their surface and exhibit enhanced pSTAT3 activation and elevated LAP/LAP* C/EBPβ isoforms, cellular mechanisms known to promote myeloid differentiation and granulocytic production. In conclusion, the switch from CD201+ to CD201- HSCs facilitates both fast and sustained emergency granulopoiesis by employing two distinct mechanisms, leading to the supply of new granulocytes to fight the infection. These observations expand our understanding on emergency granulopoiesis, which so far was understood as a process in which TLR4 expression in hematopoietic cells was dispensable (PMID: 22586037). These results have been included in Figure 6 and Results (page 13-15), and discussed in the Discussion section (page 20-21).

Next, to further explore the molecular pathway mediating CD201 downregulation downstream of LPS/inflammatory signaling, we investigated whether *Cebpb* knockout mice were able to downregulate CD201 levels upon LPS administration. Thus, WT and *Cebpb* knockout mice were injected once with LPS or PBS control, sacrificed 4 hours later, and the levels of CD201 in HSCs assessed by flow cytometric analysis. Interestingly, we observed that *Cebpb* deficient mice were able to efficiently downregulate CD201 expression in HSCs, indicating that C/EBPβ is dispensable to mediate CD201 downregulation upon LPS administration and that alternative mechanisms are responsible for this initial step during emergency granulopoiesis at the HSC level. Since C/EBPβ is mostly induced in an indirect manner during emergency granulopoiesis, we next assessed the contribution of direct pathogen sensing to CD201 downregulation. Because LPS is sensed and signals through the TLR4/MyD88 signaling pathway, we challenged MyD88 deficient mice with LPS and analyzed them similarly. Indeed, we observed that the downregulation of CD201 was diminished in MyD88 KO mice, however not to the full extent of WT mice. These results suggest that the downregulation of CD201 is partially dependent on the direct sensing of LPS by the TLR4 and the downstream MyD88-NFkB signaling, but that other factors also play a role in this process. This mechanistic insights into the downregulation of CD201 on HSC in emergency granulopoiesis have been included in a new Figure 2, the results (page 9-10), and discussion section (pages 17-18).

2. While it is clear that CD201+ HSC generate CD201- cells upon transplantation and LPS treatment, whether CD201- HSCs can regenerate the CD201+ HSC pool at later time points post LPS treatment

should be determined. For this purpose transplantation of CD201- HSCs followed by LPS treatment should be preformed.

As suggested by the reviewer CD201- HSCs were transplanted into lethally irradiated recipient mice. Engraftment and reconstitution were allowed for 16 weeks, and after, mice were subject to LPS administration to induce emergency granulopoiesis. Interestingly, we observed that transplantation of CD201- HSCs generated CD201- as well as CD201+ HSCs 16 weeks after transplant. Further, upon LPS challenge CD201 levels were reduced and the mice responded to emergency granulopoiesis. These results challenge the current use of CD201 as a HSC marker, since we observe that even CD201- HSCs are able to fully reconstitute recipient mice, produce CD201+ HSCs, and efficiently execute emergency granulopoiesis. These results have been included in Figure 4L, Figure 5, the results (page 11) and discussed in the Discussion section (page 19).

Further, we also observed that while CD201 expression is reduced at early time points upon LPS administration, 24 hour after the challenge a CD201+ HSC population reappears. Nevertheless, whether these cells are newly produced from CD201- HSC or whether they are the remained CD201+ cells expanding remains currently unknown. These results have been included in Supplementary Figure 2B.

3. Ly-HSC and My-HSC have been previously defined based on CD150 expression levels (PMID: 20304793). How this relates with CD201 expression should be determined.

We would like to thank the reviewer for suggesting this correlation. Based on previous publications and our observations, one would expect that expression of CD150 and CD201 are inversely correlated, which would support either their lymphoid or myeloid bias. However, the data revealed a more complex scenario: we observed that CD150- HSCs can be sub-divided in CD201- and CD201+ HSCs, and similarly, CD150+ HSCs can be sub-divided in CD201- and CD201+ HSCs. It would be interesting to sort these 4 distinct populations and perform transplantation assays, or perform in vivo lineage tracing experiments, to identify their fate and lineage bias. Unfortunately, these functional experiments are out of the scope of the current manuscript. The data on CD150 and CD201 expression on HSCs has been included in the results section (page 11), Figure S4F, and has been discussed in the Discussion (page 19).

4. To increase the significance of the study, the contribution of CD201+ HSC to myelopoiesis should be performed in additional settings, particularly in a in vivo infection model. The data concerning G-CSF-mediated downregulation of CD201 expression in HSCs is insuficient (S2C and S2D).

As suggested by the reviewer, we challenged mice with *Candida Albicans* to induce emergency granulopoiesis (PMID:29567783). We observed that this *in vivo* infection model also caused the lymphoid to myeloid HSC switch, which could be tracked by the loss of CD201. This result has been included in Supplementary Figure 3 and supports that our observations represent a general phenomenon during emergency granulopoiesis.

Minor concerns:
1. The absolute number of HSCs after LPS exposure should be shown.

The absolute number of HSCs after LPS exposure has been included in Supplementary Figure 1D, and shows no changes in HSC numbers 4 hours after LPS administration.

2. On page 7 I believe figure calling should read "2H-2J" instead of "3H-3J".

The Figure number has been corrected.

Referee #2:

Well-written and concise manuscript that provides relevant and novel observations on HSC biology: 1) CD201 (Procr) marks a population enriched in lymphoid-biased HSCs 2) Emergency granulopoiesis is characterized by a myeloid transcriptional switch in HSC and loss of CD201.

These observations have important implications as 1) Procr is currently considered a true HSC marker and 2) The myeloid switch that happens during emergency granulopoiesis within the HSC compartment encompasses the lymphoid-biased HSC compartment.

The experimental design is adequate and the methods state-of-the-art. The authors provide solid results with data that match the conclusions. The major limitation of this study is the lack of mechanistic insights.

There are a couple of major questions that would be critical to address to realize the full potential of the work, and other more minor aspects.

Specific major points:

1. The authors do not comment on it but it is surprising to see the virtually absent myeloid-biased HSC under PBS conditions. How do their annotated clusters and gene signatures compare to previously published observations?

For the initial annotation of clusters, we used gene expression of individual multipotent progenitor subpopulation previously published by Pietras at al. (PMID: 26095048), which separated the HSC to distinct lineage biases. Next, for the final analysis presented in the manuscript, we further defined and generated our own gene signatures of individual lineage-biases based on the initial analysis and published literature (PMID: 29198940, 29282309, 22289892, 23791645, 18674933, 24444745, 27542215).

Nevertheless, we agree with the reviewer that the lack of a myeloid-biased HSCs cluster under PBS conditions is surprising. Yet, in human bone marrow cells it was previously reported that a clear separation into single lineages was only observed among cells in the Lin−CD34+CD38+ compartment, when differentiation has progressed to the level of restricted progenitors (PMID: 28319093). Further, we identified two distinct populations (#1 and #2) that transcriptionally represent a megakaryocyte-erythroid lineage bias. Interestingly, it was previously reported that megakaryocyte/erythroid-biased multipotent progenitors are able to produce myeloid cells under basal situations (PMID: 26095048), allowing us to speculate that a similar cell fate can take place in HSCs under PBS conditions. In conclusion, we think this point is relevant and we have included it in the Discussion section (page 17).

2. How is LPS or G-CSF connected to the reduction in CD201? I understand the hurdles and limitations of performing functional studies targeting HSCs specifically, but some mechanistic insights would be required. Does Cebpb reduce the expression of CD201 in HSCs?

We thank the reviewer for this comment, which prompted us to investigate whether *Cebpb* knockout mice were able to downregulate CD201 levels upon LPS administration. Thus, WT and *Cebpb* knockout mice were injected once with LPS or PBS control, sacrificed 4 hours later, and the levels of CD201 in HSCs assessed by flow cytometric analysis. Interestingly, we observed that *Cebpb* deficient mice were able to efficiently downregulate CD201 expression in HSCs, indicating that C/EBPβ is dispensable to mediate CD201 downregulation upon LPS administration and that alternative mechanisms are responsible for this initial step during emergency granulopoiesis at the HSC level.

Since C/EBPβ is mostly induced in an indirect manner during emergency granulopoiesis, we next assessed the contribution of direct pathogen sensing to CD201 downregulation. Because LPS is sensed and signals through the TLR4/MyD88 signaling pathway, we challenged MyD88 deficient mice with LPS and analyzed them 4 hours later. Indeed, we observed that the downregulation of CD201 was diminished in MyD88 KO mice, however not to the full extent of WT mice. These results suggest that the downregulation of CD201 is partially dependent on the direct sensing of LPS by the TLR4 and the downstream MyD88-NFkB signaling, but that other factors also play a role in this process.

This mechanistic insights into the downregulation of CD201 on HSC in emergency granulopoiesis have been included in a new Figure 2, the results (page 9-10), and discussion section (pages 17-18).

3. Is the loss of CD201 consequence or can it also contribute to the lymphoid to myeloid transcriptional switch? To rule out this question, the authors may want to perform a short term in vivo treatment with a blocking antibody.

We thank the reviewer for this suggestion and performed the proposed experiment which revealed that the CD201 blocking antibody partially impairs the lymphoid to myeloid switch. Precisely, mice were treated with CD201 function-blocking antibody or PBS control as previously described (PMID: 32294155), and 24 hours later they received an LPS injection. Mice were sacrificed 4 hours later and emergency granulopoiesis at the stem and progenitor level was assessed. We observed that upon LPS stimulation mice that received one injection of CD201 blocking antibody exhibited hallmarks of emergency granulopoiesis, however, the response was significantly lower than in non-blocking antibody treated control mice. Altogether, this experiment suggests that CD201 functionally contributes to the lymphoid to myeloid switch. This observation and the corresponding conclusions have been included in the manuscript (Figure 5A-B and results Page 12).

4. Which subset CD201+ HSC or CD201- HSC performs better under emergency granulopoiesis or are they just the same? Are the dynamics of the process similar for both? The authors show a different response for MPP3 but more information would be relevant. Are they both really needed to respond under emergency granulopoiesis? One may think that a more adaptive response would involve increased granulocyte output without compromising the lymphoid output.

We thank the reviewer for this remark which together with the previous points and the comments from reviewer 1 helped us to gain important knowledge on the mechanistic insight that drives emergency granulopoiesis at the HSC level.

We hypothesized that emergency granulopoiesis might be activated in HSCs in a direct (LPS/TLR4) or indirect (G-CSF/G-CSF-R) manner. Of note, we observed that G-CSF levels are greatly increased 4 hours upon LPS injection, indicating that both activating mechanisms are potentially involved in the process. Thus, we investigated the mechanism driving emergency granulopoiesis on CD201+ and CD201- HSCs. Interestingly, we observed that both CD201+ and CD201- HSCs rely on different signaling pathways under acute infection. While CD201+ HSCs express higher levels of TLR4 and have higher activation of NF-κB signaling upon *in vitro* LPS stimulation, the CD201- HSCs express higher levels of G-CSF-R and a have higher activation of pSTAT3 signaling upon G-CSF-stimulation. Moreover, while the LIP isoform of C/EBPβ (important for cell proliferation) is present in both CD201+ and CD201- HSCs, the LAP/LAP* C/EBPβ isoforms (important for myeloid differentiation) are present only in the CD201- HSCs fraction.

If we incorporate these results to our previous observations, we conclude that emergency granulopoiesis is supported by a subpopulation of HSCs which upon pathogen sensing undergo a radical transcriptional rewiring that promotes their myeloid output. Initially, the pathogen is directly sensed by TLR4 on the surface of a steady state lymphoid-biased CD201+ HSCs, causing a rapid activation of the downstream NF-κB signaling pathway. Subsequently, the lymphoid-myeloid transcriptional switch, marked by the loss of CD201 expression, occurs and emergency granulopoiesis is then supported by myeloid-biased CD201- HSCs. CD201- HSCs respond to the infection in an indirect manner through G-CSF-R on their surface and exhibit enhanced pSTAT3 activation and elevated LAP/LAP* C/EBPβ isoform, cellular mechanisms known to promote myeloid differentiation and granulocytic production. In conclusion, the switch from CD201+ to CD201- HSCs facilitates both fast and sustained emergency granulopoiesis by employing two distinct mechanisms, leading to the supply of new granulocytes to fight the infection. These observations expand our understanding on emergency granulopoiesis, which so far was understood as a process in which TLR4 expression in hematopoietic cells was dispensable (PMID: 22586037). These results have been included in Figure 6 and Results (page 13-15), and discussed in the Discussion section (page 20-21).

5. Do lymphoid-biased HSC regain CD201 expression under resting conditions following LPS challenge?

We also observed that while CD201 expression is reduced at early time points upon LPS administration, 24 hour after the challenge a CD201+ HSC population reappears. Nevertheless, whether these cells are newly produced from CD201- HSC or whether they are the remained CD201+ cells expanding remains currently unknown. These results have been included in Supplementary Figure 2B.

Specific minor points:

1. Figures 2H-2J are referenced in the text as 3H-3J. Please correct.

The Figure number has been corrected.

2. Under my opinion, study of the preferential lineage output of CD201+ HSC in vivo "under steady state conditions" would require lineage tracing. I suggest to rephrase the experiments using lethal irradiation and transplantation to "resting conditions following hematopoietic challenge" or similar.

We thank the reviewer for this remark which provides a more accurate definition of the conditions. We adapted the text accordingly.

3. Please provide a brief explanation of the pseudo-time analysis in the Results section.

As suggested we included on page 7 a sentence clarifying that pseudo-time analysis places cells onto a linear trajectory following a continuum of gene expression changes of cellular states.

4. Please reference Table S1 for better understanding of Figure 1H.

The reference has been included.

5. Indicate if the mice are SOPF, especially relevant in studies involving inflammation and myelopoiesis.

Indeed, mice were kept under specific pathogen free conditions. We apologize for omitting this information, which has been now included in the Material and methods section under Animal models.

6. Are CD201- HSC able to generate CD201+ HSC?

To address this point, we transplanted CD201- HSCs into lethally irradiated recipient mice. Engraftment and reconstitution were allowed for 16 weeks. Interestingly, we observed that transplantation of CD201- HSCs generated CD201- as well as CD201+ HSCs 16 weeks after transplant. These results challenge the current use of CD201 as a HSC marker, since we observe that even CD201- HSCs are able to fully reconstitute recipient mice and produce CD201+ HSCs. These results have been included in Figure 4L, results section (page 11), and discussed in the Discussion section (page 19).

7. Fig 1I, please show UMAP of both PBS and LPS separately as well. From this panel, it seems like CD201+ cells comprise a small subset within the lymphoid-biased HSC cluster. Please discuss.

With respect to separate visualization for PBS and LPS conditions in Fig 1I, the UMAP was calculated for both conditions together. Recalculation for each condition separately would change the UMAP topology and make the visualizations not comparable. We would like to point the reviewer to UMAP in Figure 1B, where it is visible that most of the cells in cluster 3 are from the PBS treated sample. Note that in Figure 1B the separation based on sample type was possible, because in that case relative gene expression was not calculated.

With respect to the abundance of CD201+ HSCs, we have followed the expression of Procr/CD201 with single cell resolution by (i) scRNA-seq at the level of gene expression and (ii) at the level of protein by FACS. The FACS data, which is more quantitative, is restricted to the analysis of CD201 levels, and shows that CD201 expression forms a continuum in the HSC population and not a discrete subset. On the other hand, in scRNAseq data, UMAP and cluster assignments are based on the analysis of the expression of multiple genes, yet the visualization has the limitation that each individual gene, even a highly expressed one, will randomly be detected only in a subset of cells of a given (intrinsic limitation of scRNAseq). However, despite the discrete representation of CD201 expression on the UMAP, *Procr* is one of the top-

ranking marker genes of cluster 3 (see Supplementary Table 1). And this is supported by the levels of expression and percentage of HSCs expressing CD201 which can be properly and more accurately quantified by the FACS analysis. This has been clarified in the results section Page 9.

8. It would be preferable to use FACS gating strategies with lymphoid and myeloid cell subsets separatetely.

While we agree that using independent gating strategies to identify the presence of B-, T-, and myeloid cells is an option, it does not allow us to compare all 3 lineages in one plot. Using this strategy, contribution to each lineage can be easily visualized looking at one plot and quantified as percentage (being the total 100%). Unfortunately, due to the fluorochrome combination this strategy does not allow to separate the lymphoid and myeloid cell subsets in separate plots.

1st Revision - Editorial Decision 15th Sep 2023

Dear Meritxell,

Thank you for submitting your revised version. Your study has now been seen by the original referees and as you can see below, they appreciate the introduced revisions. They have a few remaining points that shouldn't be too much work to sort out. I am therefore very pleased to accept the manuscript for publication here pending minor revisions. When you submit your revised version will you also take care of the following editorial points.:

- We need 3-5 keywords

- REFERENCE FORMAT: should be 10 authors + et al. Currently it is 20 authors et al.

- COI needs renaming to "DISCLOSURE AND COMPETING INTERESTS STATEMENT"

- Please remove the Authors Contributions from the manuscript. The 'Author Contributions' section is replaced by the CRediT contributor roles taxonomy to specify the contributions of each author in the journal submission system. Please use the free text box in the 'author information' section of the manuscript submisssion system to provide more detailed descriptions (e.g., 'X provided intracellular Ca++ measurements in fig Y')

- The appendix file is missing a ToC with page numbers. Nomenclature should be Appendix Figure S1-S4 with the corresponding callouts.

- Supplementary Tables should be renamed to Table EV1-EV2 with the appropriate callouts, or included in Appendix PDF and renamed to Appendix Table S1-S2

- Figure legends should be placed after References

- Author email bounced for Petr Danek and Monika Burocziova

- Our publisher has also done their pre-publication check on your manuscript. When you log into the manuscript submission system you will see the file "Data Edited Manuscript file". Please look at the word file and the comments regarding the figure legends and respond to the issues.

- Please upload a synopsis text. We need a summary statement plus 3-5 bullet points describing the key findings of the MS.

- We also need a synopsis image => 550 wide by [200-400]

That should be all! Congratulations on a nice study

With best wishes

Karin

Karin Dumstrei, PhD
Senior Editor
The EMBO Journal

Instructions for preparing your revised manuscript:

Guide For Authors: https://www.embopress.org/page/journal/14602075/authorguide

Use the link below to submit your revision:

https://emboj.msubmit.net/cgi-bin/main.plex

Referee #1:

In the revised version of their study, Vanickova et al. have made a substantial effort to improve the overall quality of the

manuscript. They have experimentally addressed the key questions raised by the reviewers and provide evidence for the following major points:

1) Molecular pathways acting in CD201+ and CD201- HSCs during emergency granulopoiesis: authors show that while CD201+ HSCs express higher levels of TLR4 and NFkB activation (on this point please see major concern bellow) upon LPS stimulation, suggesting a direct sensing of LPS, while CD201- HSCs express higher levels of G-CSF receptor, increased pSTAT3 levels and elevated LAP/LAP* C/EBPB isoforms in response to LPS, suggestive of an indirect sensing of LPS (via G-CSF) and delayed granulopoiesis activation.

2) Molecular pathways implicated in LPS-mediated downregulation of CD201 during emergency granulopoiesis: authors show that while Cebpb is dispensable for LPS-mediated downregulation of CD201, MyD88 is involved in this process.

Concerning point 1, it might be my own misunderstand but as far as I know, higher levels of phosphorylated IkBA indicate higher levels of NFkB activation, since IkBA is a NFkB inhibitor that is marked for degradation upon phosphorylation. Thus the interpretation of graph 6B is incorrect, as CD201+ have lower pIkBA and consequently lower NFkB activation. This needs to be clarified as it impacts on the current data interpretation.

Overall, upon addressing the previously addressed point, I believe the revised manuscript to be of merit for publication in EMBO journal.

Referee #2:

The authors have done a remarkable job addressing my previous concerns. The extensive revisions provide convincing molecular insights into the downregulation as well as the functional contribution of CD201 in emergency granulopoiesis, and the selective roles of CD201+ and CD201- HSCs in the process. The authors further point out to differential molecular mechanisms mediating these selective roles.

I have some comments related to new figure 6:

-Panels A,B,D,E - I suggest replacement by bar plots consistent with the rest of the paper. As they are now, the lines could be wrongly interpreted as kinetic plots at a first glance.

-Mechanistic studies in panels B,E - Numbers are low to make strong conclusions, so it is advisable to increase the numbers. If not possible, please tone down the conclusions.
We are missing the basal measurement for a full understanding of the result. Providing the results relative to basal as fold change would also be an option, consistent with the rest of the results in the manuscript.

-Differences in p-IkBa and pSTAT3 between CD201+ and CD201- HSC seem, as they are now, rather small. Given that the authors provide no functional studies showing direct evidence on their roles (which is out of the scope of the manuscript at this point), it would be relevant to show the activation status of both for LPS and G-CSF conditions, as well as the activation status of other potentially relevant downstream components of MyD88 pathway (i.e. MAP3K - AP-1) and of G-CSF-R (PI3K - AKT; ERK). These studies would help strengthen their conclusions.

-Is the elevation of LAP/LAP* C/EBPB isoforms connected to the activation of G-CSF-R in CD201- HSC?

Finally, I would like to congratulate the authors on this interesting work!

First of all, we would like to thank the reviewers for their comments and the time they invested in reviewing our paper. We believe that their input definitively helped us to make our manuscript solid and interesting. Here, we address the remaining few points.

Referee #1:

In the revised version of their study, Vanickova et al. have made a substantial effort to improve the overall quality of the manuscript. They have experimentally addressed the key questions raised by the reviewers and provide evidence for the following major points:

1) Molecular pathways acting in CD201+ and CD201- HSCs during emergency granulopoiesis: authors show that while CD201+ HSCs express higher levels of TLR4 and NFkB activation (on this point please see major concern bellow) upon LPS stimulation, suggesting a direct sensing of LPS, while CD201- HSCs express higher levels of G-CSF receptor, increased pSTAT3 levels and elevated LAP/LAP* C/EBPB isoforms in response to LPS, suggestive of an indirect sensing of LPS (via G-CSF) and delayed granulopoiesis activation.

2) Molecular pathways implicated in LPS-mediated downregulation of CD201 during emergency granulopoiesis: authors show that while Cebpb is dispensable for LPS-mediated downregulation of CD201, MyD88 is involved in this process.

Concerning point 1, it might be my own misunderstand but as far as I know, higher levels of phosphorylated IkBA indicate higher levels of NFkB activation, since IkBA is a NFkB inhibitor that is marked for degradation upon phosphorylation. Thus the interpretation of graph 6B is incorrect, as CD201+ have lower pIkBA and consequently lower NFkB activation. This needs to be clarified as it impacts on the current data interpretation.

Overall, upon addressing the previously addressed point, I believe the revised manuscript to be of merit for publication in EMBO journal.

We thank the reviewer for the positive feed-back, specially for noticing the mislabeling in Figure 6B. The reviewer is correct, as phosphorylated IkBA is targeted for ubiquitin-dependent degradation, allowing p50/p65 to translocate to the nucleus and leading to NFkB activation. Figure 6B should say IKBA (not p-IKBA). However, to be totally sure it was just a mislabeling, we run the experiment again making sure we are using an antibody against total IKBA. Indeed, IkBA levels are higher in CD201-HSCs than in CD201+ HSC, which would indicate higher NFkB activation in the CD201+ HSCs. Additionally, we also assessed p65 levels, and observed that they are higher in CD201+ HSCs, poiting to a higher NFkB activity in these cells.

Referee #2:

The authors have done a remarkable job addressing my previous concerns. The extensive revisions provide convincing molecular insights into the downregulation as well as the functional contribution of CD201 in emergency granulopoiesis, and the selective roles of CD201+ and CD201- HSCs in the process. The authors further point out to differential molecular mechanisms mediating

these selective roles.

I have some comments related to new figure 6:

-Panels A,B,D,E - I suggest replacement by bar plots consistent with the rest of the paper. As they are now, the lines could be wrongly interpreted as kinetic plots at a first glance.

In this particular set of experiments, we are comparing CD201+ to CD201- HSCs isolated from the same mouse. We have tried visualizing the results as bar plots as in the rest of the paper, but then we don't see the pairs and the results are less visual. We believe that the current representation is more visual and accurate for these type of data sets.

-Mechanistic studies in panels B,E - Numbers are low to make strong conclusions, so it is advisable to increase the numbers. If not possible, please tone down the conclusions.
We are missing the basal measurement for a full understanding of the result. Providing the results relative to basal as fold change would also be an option, consistent with the rest of the results in the manuscript.

We agree that the numbers are low, and we have increased the number of samples to make the results stronger. Please refer to new Figure 6B and E which both now include the analysis of 12 mice (one data point represents a pool of HSCs sorted from 2 mice).

In this panels we decided not to show the differences relative to the basal levels because that would require the use of many more animals. These phospho-flow experiments are performed on sorted and CD201+ and CD201- HSCs, followed by intracellular staining, overnight incubations, and several washing steps. Consequently, to sort enough cells we need large number of animals which need to be processed and sorted in a time sensitive manner not to compromise cell viability. Thus, to run the basal activity together with the stimulated condition, would be technically challenging and could compromise the viability of our cells and the results.

-Differences in p-IkBa and pSTAT3 between CD201+ and CD201- HSC seem, as they are now, rather small. Given that the authors provide no functional studies showing direct evidence on their roles (which is out of the scope of the manuscript at this point), it would be relevant to show the activation status of both for LPS and G-CSF conditions, as well as the activation status of other potentially relevant downstream components of MyD88 pathway (i.e. MAP3K - AP-1) and of G-CSF-R (PI3K - AKT; ERK). These studies would help strengthen their conclusions.

Indeed, we agree with the reviewer that additional signaling molecules could be analyzed. But unfortunately, due to the limited number of HSCs we can sort from one mouse, we feel that at this stage this goes beyond the present manuscript. This would require multiple rounds of optimization to determine the right timepoint of stimulation for these individual target molecules. Nevertheless, we have included p65 levels in panel 6B, which would support enhanced NF-KB activation in the CD201+ HSCs.

-Is the elevation of LAP/LAP* C/EBPB isoforms connected to the activation of G-CSF-R in CD201- HSC?

We thank the reviewer for raising this interesting point. The data presented in Figure 6G was obtained using non-stimulated cells, which would suggest that the LAP/LAP elevation is present in basal conditions. Nevertheless, we cannot exclude that the elevation is mediated in vivo and we are*

in fact assessing the response to basal G-CSF levels in mice. In addition, we also assessed LAP/LAP levels upon G-CSF stimulation ex vivo, and we indeed observe a tendency to increase LAP/LAP* upon G-CSF stimulation. Nevertheless, we think that the LAP/LAP* regulation by G-CSF/G-CSF-R axis is dose and time dependent and we would need additional experiments to make this statement ready for publication.*

Finally, I would like to congratulate the authors on this interesting work!

2nd Revision - Editorial Decision 25th Sep 2023

Dear Meritxell,

Thank you for submitting your revised manuscript to The EMBO Journal. I have now looked at everything and all looks good!

I am therefore very pleased to accept the manuscript for publication here.

Congratulations on a nice study

Best Karin

Karin Dumstrei, PhD
Senior Editor
The EMBO Journal

Please note that it is EMBO Journal policy for the transcript of the editorial process (containing referee reports and your response letter) to be published as an online supplement to each paper. If you do NOT want this, you will need to inform the Editorial Office via email immediately. More information is available here:
https://www.embopress.org/page/journal/14602075/authorguide#transparentprocess

Your manuscript will be processed for publication in the journal by EMBO Press. Manuscripts in the PDF and electronic editions of The EMBO Journal will be copy edited, and you will be provided with page proofs prior to publication. Please note that supplementary information is not included in the proofs.

You will be contacted by Wiley Author Services to complete licensing and payment information. The required 'Page Charges Authorization Form' is available here: https://www.embopress.org/pb-assets/embo-site/tej_apc.pdf - please download and complete the form and return to embopressproduction@wiley.com

EMBO Press participates in many Publish and Read agreements that allow authors to publish Open Access with reduced/no publication charges. Check your eligibility: https://authorservices.wiley.com/author-resources/Journal-Authors/open-access/affiliation-policies-payments/index.html

Should you be planning a Press Release on your article, please get in contact with embojournal@wiley.com as early as possible, in order to coordinate publication and release dates.

If you have any questions, please do not hesitate to call or email the Editorial Office. Thank you for your contribution to The EMBO Journal.

** Click here to be directed to your login page: https://emboj.msubmit.net

EMBO Press Author Checklist

Corresponding Author Name: Meritxell Alberich Jorda
Journal Submitted to: The EMBO Journal
Manuscript Number: EMBOJ-2023-113527R

USEFUL LINKS FOR COMPLETING THIS FORM
The EMBO Journal - Author Guidelines
EMBO Reports - Author Guidelines
Molecular Systems Biology - Author Guidelines
EMBO Molecular Medicine - Author Guidelines

Reporting Checklist for Life Science Articles (updated January
This checklist is adapted from Materials Design Analysis Reporting (MDAR) Checklist for Authors. MDAR establishes a minimum set of requirements in transparent reporting in the life sciences (see Statement of Task: 10.31222/osf.io/9sm4x). Please follow the journal's guidelines in preparing your
Please note that a copy of this checklist will be published alongside your article.

Abridged guidelines for figures
1. Data
The data shown in figures should satisfy the following conditions:
- the data were obtained and processed according to the field's best practice and are presented to reflect the results of the experiments in an accurate and unbiased manner.
- ideally, figure panels should include only measurements that are directly comparable to each other and obtained with the same assay.
- plots include clearly labeled error bars for independent experiments and sample sizes. Unless justified, error bars should not be shown for technical
- if n<5, the individual data points from each experiment should be plotted. Any statistical test employed should be justified.
- Source Data should be included to report the data underlying figures according to the guidelines set out in the authorship guidelines on Data

2. Captions
Each figure caption should contain the following information, for each panel where they are relevant:
- a specification of the experimental system investigated (eg cell line, species name).
- the assay(s) and method(s) used to carry out the reported observations and measurements.
- an explicit mention of the biological and chemical entity(ies) that are being measured.
- an explicit mention of the biological and chemical entity(ies) that are altered/varied/perturbed in a controlled manner.
- the exact sample size (n) for each experimental group/condition, given as a number, not a range;
- a description of the sample collection allowing the reader to understand whether the samples represent technical or biological replicates (including how many animals, litters, cultures, etc.).
- a statement of how many times the experiment shown was independently replicated in the laboratory.
- definitions of statistical methods and measures:
  - common tests, such as t-test (please specify whether paired vs. unpaired), simple χ2 tests, Wilcoxon and Mann-Whitney tests, can be unambiguously identified by name only, but more complex techniques should be described in the methods section;
  - are tests one-sided or two-sided?
  - are there adjustments for multiple comparisons?
  - exact statistical test results, e.g., P values = x but not P values < x;
  - definition of 'center values' as median or average;
  - definition of error bars as s.d. or s.e.m.

Please complete ALL of the questions below.
Select "Not Applicable" only when the requested information is not relevant for your study.

Materials

Newly Created Materials	Information included in the manuscript?	In which section is the information available? (Reagents and Tools Table, Materials and Methods, Figures, Data Availability Section)
New materials and reagents need to be available; do any restrictions apply?	Not Applicable	

Antibodies	Information included in the manuscript?	In which section is the information available? (Reagents and Tools Table, Materials and Methods, Figures, Data Availability Section)
For **antibodies** provide the following information:<br>- Commercial antibodies: RRID (if possible) or supplier name, catalogue number and or/clone number<br>- Non-commercial: RRID or citation	Yes	Materials and Methods

DNA and RNA sequences	Information included in the manuscript?	In which section is the information available? (Reagents and Tools Table, Materials and Methods, Figures, Data Availability Section)
**Short novel DNA or RNA including primers, probes**: provide the sequences.	Yes	Materials and Methods

Cell materials	Information included in the manuscript?	In which section is the information available? (Reagents and Tools Table, Materials and Methods, Figures, Data Availability Section)
**Cell lines:** Provide species information, strain. Provide accession number in repository **OR** supplier name, catalog number, clone number, and/**OR** RRID.	Not Applicable	
**Primary cultures:** Provide species, strain, sex of origin, genetic modification status.	Yes	Materials and Methods
Report if the cell lines were recently **authenticated** (e.g., by STR profiling) and tested for mycoplasma contamination.	Not Applicable	

Experimental animals	Information included in the manuscript?	In which section is the information available? (Reagents and Tools Table, Materials and Methods, Figures, Data Availability Section)
**Laboratory animals or Model organisms:** Provide species, strain, sex, age, genetic modification status. Provide accession number in repository **OR** supplier name, catalog number, clone number, **OR** RRID.	Yes	Materials and Methods
**Animal observed in or captured from the field:** Provide species, sex, and age where possible.	Not Applicable	
Please detail **housing and husbandry conditions**.	Yes	Materials and Methods

Plants and microbes	Information included in the manuscript?	In which section is the information available? (Reagents and Tools Table, Materials and Methods, Figures, Data Availability Section)
**Plants:** provide species and strain, ecotype and cultivar where relevant, unique accession number if available, and source (including location for collected wild specimens).	Not Applicable	
**Microbes:** provide species and strain, unique accession number if available, and source.	Yes	Materials and Methods

Human research participants	Information included in the manuscript?	In which section is the information available? (Reagents and Tools Table, Materials and Methods, Figures, Data Availability Section)
If collected and within the bounds of privacy constraints report on age, sex and gender or ethnicity for all study participants.	Not Applicable	

Core facilities	Information included in the manuscript?	In which section is the information available? (Reagents and Tools Table, Materials and Methods, Figures, Data Availability Section)
If your work benefited from core facilities, was their service mentioned in the acknowledgments section?	Yes	Acknowledgements

Design

Study protocol	Information included in the manuscript?	In which section is the information available? (Reagents and Tools Table, Materials and Methods, Figures, Data Availability Section)
If study protocol has been **pre-registered, provide DOI in the manuscript**. For clinical trials, provide the trial registration number **OR** cite DOI.	Not Applicable	
Report the **clinical trial registration number** (at ClinicalTrials.gov or equivalent), where applicable.	Not Applicable	

Laboratory protocol	Information included in the manuscript?	In which section is the information available? (Reagents and Tools Table, Materials and Methods, Figures, Data Availability Section)
Provide DOI OR other citation details if **external detailed step-by-step protocols** are available.	Yes	Materials and Methods

Experimental study design and statistics	Information included in the manuscript?	In which section is the information available? (Reagents and Tools Table, Materials and Methods, Figures, Data Availability Section)
Include a statement about **sample size** estimate even if no statistical methods were used.	Yes	Figure legends
Were any steps taken to minimize the effects of subjective bias when allocating animals/samples to treatment (e.g. **randomization procedure**)? If yes, have they been described?	Not Applicable	
Include a statement about **blinding** even if no blinding was done.	Yes	Materials and Methods
Describe **inclusion/exclusion criteria** if samples or animals were excluded from the analysis. Were the criteria pre-established?<br><br>If sample or data points were omitted from analysis, report if this was due to attrition or intentional exclusion and provide justification.	Yes	Materials and Methods
For every figure, are **statistical tests** justified as appropriate? Do the data meet the assumptions of the tests (e.g., normal distribution)? Describe any methods used to assess it. Is there an estimate of variation within each group of data? Is the variance similar between the groups that are being statistically compared?	Yes	Figure legends and Materials and Methods

Sample definition and in-laboratory replication	Information included in the manuscript?	In which section is the information available? (Reagents and Tools Table, Materials and Methods, Figures, Data Availability Section)
In the figure legends: state number of times the experiment was **replicated** in laboratory.	Yes	Figure legends
In the figure legends: define whether data describe **technical or biological replicates**.	Yes	Figure legends

Ethics

Ethics	Information included in the manuscript?	In which section is the information available? (Reagents and Tools Table, Materials and Methods, Figures, Data Availability Section)
Studies involving **human participants**: State details of **authority granting ethics approval** (IRB or equivalent committee(s), provide reference number for approval.	Not Applicable	
Studies involving **human participants**: Include a statement confirming that **informed consent** was obtained from all subjects and that the experiments conformed to the principles set out in the WMA Declaration of Helsinki and the Department of Health and Human Services Belmont Report.	Not Applicable	
Studies involving **human participants:** For publication of **patient photos**, include a statement confirming that consent to publish was obtained.	Not Applicable	
Studies involving experimental **animals**: State details of **authority granting ethics approval** (IRB or equivalent committee(s), provide reference number for approval. Include a statement of compliance with ethical regulations.	Yes	Materials and Methods
Studies involving **specimen and field samples:** State if relevant **permits** obtained, provide details of authority approving study; if none were required, explain why.	Not Applicable	

Dual Use Research of Concern (DURC)	Information included in the manuscript?	In which section is the information available? (Reagents and Tools Table, Materials and Methods, Figures, Data Availability Section)
Could your study fall under dual use research restrictions? Please check biosecurity documents and list of **select agents and toxins** (CDC): https://www.selectagents.gov/sat/list.htm	Not Applicable	
If you used a select agent, is the security level of the lab appropriate and reported in the manuscript?	Not Applicable	
If a study is subject to dual use research of concern regulations, is the name of the **authority granting approval and reference number** for the regulatory approval provided in the manuscript?	Not Applicable	

Reporting

The MDAR framework recommends adoption of discipline-specific guidelines, established and endorsed through community initiatives. Journals have their own policy about requiring specific guidelines and recommendations to complement MDAR.

Adherence to community standards	Information included in the manuscript?	In which section is the information available? (Reagents and Tools Table, Materials and Methods, Figures, Data Availability Section)
State if relevant guidelines or checklists (e.g., **ICMJE, MIBBI, ARRIVE, PRISMA**) have been followed or provided.	Not Applicable	
For **tumor marker prognostic studies,** we recommend that you follow the **REMARK** reporting guidelines (see link list at top right). See author guidelines, under 'Reporting Guidelines'. Please confirm you have followed these guidelines.	Not Applicable	
For **phase II and III randomized controlled trials**, please refer to the **CONSORT** flow diagram (see link list at top right) and submit the CONSORT checklist (see link list at top right) with your submission. See author guidelines, under 'Reporting Guidelines'. Please confirm you have submitted this list.	Not Applicable	

Data Availability

Data availability	Information included in the manuscript?	In which section is the information available? (Reagents and Tools Table, Materials and Methods, Figures, Data Availability Section)
Have **primary datasets** been deposited according to the journal's guidelines (see 'Data Deposition' section) and the respective accession numbers provided in the Data Availability Section?	Yes	Data availability section
Were **human clinical and genomic datasets** deposited in a public access-controlled repository in accordance to ethical obligations to the patients and to the applicable consent agreement?	Not Applicable	
Are **computational models** that are central and integral to a study available without restrictions in a machine-readable form? Were the relevant accession numbers or links provided?	Not Applicable	
If publicly available data were reused, provide the respective **data citations in the reference list**.	Not Applicable	
